# NIPSNAP1 directs dual mechanisms to restrain senescence in cancer cells

**DOI:** 10.1186/s12967-023-04232-1

**Published:** 2023-06-20

**Authors:** Enyi Gao, Xiaoya Sun, Rick Francis Thorne, Xu Dong Zhang, Jinming Li, Fengmin Shao, Jianli Ma, Mian Wu

**Affiliations:** 1 Translational Research Institute, Henan Provincial People’s Hospital, School of Clinical Medicine, Henan University, Zhengzhou, 450046 China; 2 School of Basic Medical Sciences, Henan University, Zhengzhou, 450046 China; 3 School of Basic Medical Sciences, Zhengzhou University, Zhengzhou, 450001 China; 4 Henan Provincial People’s Hospital, Zhengzhou University, Zhengzhou, 450003 China; 5 Department of Radiation Oncology, Harbin Medical University Cancer Hospital, Harbin, China

**Keywords:** NIPSNAP1, Cellular senescence, FBXL14, c-Myc, ROS, SOD2, SIRT3, Acetylation

## Abstract

**Background:**

Although the executive pathways of senescence are known, the underlying control mechanisms are diverse and not fully understood, particularly how cancer cells avoid triggering senescence despite experiencing exacerbated stress conditions within the tumor microenvironment.

**Methods:**

Mass spectrometry (MS)-based proteomic screening was used to identify differentially regulated genes in serum-starved hepatocellular carcinoma cells and RNAi employed to determine knockdown phenotypes of prioritized genes. Thereafter, gene function was investigated using cell proliferation assays (colony-formation, CCK-8, Edu incorporation and cell cycle) together with cellular senescence assays (SA-β-gal, SAHF and SASP). Gene overexpression and knockdown techniques were applied to examine mRNA and protein regulation in combination with luciferase reporter and proteasome degradation assays, respectively. Flow cytometry was applied to detect changes in cellular reactive oxygen species (ROS) and in vivo gene function examined using a xenograft model.

**Results:**

Among the genes induced by serum deprivation, NIPSNAP1 was selected for investigation. Subsequent experiments revealed that NIPSNAP1 promotes cancer cell proliferation and inhibits P27-dependent induction of senescence via dual mechanisms. Firstly, NIPSNAP1 maintains the levels of c-Myc by sequestering the E3 ubiquitin ligase FBXL14 to prevent the proteasome-mediated turnover of c-Myc. Intriguingly, NIPSNAP1 levels are restrained by transcriptional repression mediated by c-Myc-Miz1, with repression lifted in response to serum withdrawal, thus identifying feedback regulation between NIPSNAP1 and c-Myc. Secondly, NIPSNAP1 was shown to modulate ROS levels by promoting interactions between the deacetylase SIRT3 and superoxide dismutase 2 (SOD2). Consequent activation of SOD2 serves to maintain cellular ROS levels below the critical levels required to induce cell cycle arrest and senescence. Importantly, the actions of NIPSNAP1 in promoting cancer cell proliferation and preventing senescence were recapitulated in vivo using xenograft models.

**Conclusions:**

Together, these findings reveal NIPSNAP1 as an important mediator of c-Myc function and a negative regulator of cellular senescence. These findings also provide a theoretical basis for cancer therapy where targeting NIPSNAP1 invokes cellular senescence.

**Supplementary Information:**

The online version contains supplementary material available at 10.1186/s12967-023-04232-1.

## Introduction

Cellular senescence, otherwise known as irreversible cell cycle arrest, is classically associated with the finite replicative lifespan of primary cells [1]. Along with ceasing proliferation, senescent cells display characteristic features including increased senescence-associated beta-galactosidase activity (SA-β-gal), the secretion of a complex array of proinflammatory cytokines called the senescence-associated secretory phenotype (SASP) together with the formation of senescence associated heterochromatin foci (SAHF) representing accumulated DNA damage [2, 3]. Nevertheless, beyond cell lifespan limitations, senescence represents a complex cellular reaction to a range of different stressors including responses to genotoxic drugs, radiation, nutrient deficiency, hypoxia, mitochondrial dysfunction, along with the effects of oncogenic signaling. Interestingly, cancer cells may respond to chemotherapy or radiotherapy treatments by entering senescence [4], popularizing the idea that inducing senescence in cancer cells as a therapeutic option [5]. However, exploiting this idea has been contentious since abnormal senescence mechanisms have been shown to contribute to the development of tumors [6, 7]. Thus, it is imperative to fully understand the pathways involved in order to harness senescence programming as therapy.

One of the key modulators of senescence is c-Myc, a proto-oncogene that is aberrantly activated in ~ 40% of human cancers. In normal and malignant cells alike, c-Myc broadly contributes to various physiological processes including the regulation of metabolism, proliferation, apoptosis, and differentiation [8]. Additionally, the dysregulation of c-Myc is associated with chromosomal translocations, gene amplifications, together with DNA insertions and mutations, all of which contribute to oncogenesis and the progression of tumors [9–11]. Notably, the overexpression of c-Myc can suppress senescence, for example in melanoma [12, 13] and moreover, various studies have shown that c-Myc can inhibit senescence through extensive connections to the regulation of cell cycle-related proteins [14–17]. It has also been shown that c-Myc activates telomerase by inducing transcription of hTERT to prevent cells from aging and even acts to immortalize cells [18]. Alternatively, reductions in c-Myc expression and activity can elicit senescence in cancer cells [12, 19]. Together, these and other studies highlight the point that c-Myc plays a multifaceted role in the regulation of senescence, although the underlying mechanisms related to different conditions, cell types and disease states likely differ.

Another key predisposing factor governing senescence is oxidative stress, with the accumulation of reactive oxygen species (ROS) capable of inducing senescence [20]. While cellular ROS homeostasis is maintained through several antioxidant systems, sustained redox imbalances may be sufficient to drive entry into senescence in a range of cell types. For example, oxidative stress in proliferating stem cells generated through oxidative phosphorylation can promote senescence [21, 22] while treatments that reduce the accumulation of ROS such as the DNA methyltransferase inhibitor 5-azacytidine which increases superoxide dismutase activity reverses the senescent phenotype of mesenchymal stem cells [23]. And in osteoarthritis, imbalances in redox homeostasis in chondrocytes impairs their capacity to synthesize selenoproteins, impairing oxidoreductase capacity and promoting chondrocyte senescence [24]. Interestingly, cancer cells universally exhibit increased ROS levels compared to normal cells with ROS signaling contributing to proliferation, survival, and adaptation to hypoxia among a variety of benefits [25, 26]. Nevertheless, cancer cells must maintain ROS levels below critical levels which could induce deleterious effects including cell death or senescence. Therefore, it is important to properly define the underlying mechanisms determining the induction of cellular senescence in tumors.

Of relevance to this report are the NIPSNAP (4-nitrophenyl phosphatase and non-neuronal SNAP25 like protein homolog) family which include NIPSNAP genes 1–4. The archetypal NIPSNAP1 has been shown to play a role in regulating synaptic activity in learning and memory [27–30] but nonetheless, along with the brain, NIPSNAP1 is highly abundant in other organs such as the liver, kidney, and adrenals but its expression is less evident in other tissues such as skeletal muscle. Each NIPSNAP protein contains an evolutionary conserved 110 amino acid domain; however, relatively little is known about the molecular function of the members of the NIPSNAP family [31]. The most influential studies to date involve the identification of NIPSNAP1 and NIPSNAP2 as binding partners of LC3/GABARAP, autophagy-associated proteins that serve to regulate autophagosomes [32, 33], with a more recent follow up report showing that NIPSNAP1/2 are mitochondrial proteins essential for mitophagy [34]. Here, we alternatively identified NIPSNAP1 in a proteomics screen seeking to identify factors essential for the survival of cancer cells subjected to serum deprivation stress, a scenario that recapitulates loss of growth factor signaling within the tumor microenvironment.

Functional investigations revealed the knockdown of NIPSNAP1 in cancer cells induced cell cycle arrest and inhibited proliferation with the underlying phenotype resulting from senescence. Further analyses revealed that NIPSNAP1 engages in two separate binding interactions that prevent cellular senescence. First, NIPSNAP1 engages with the E3 ubiquitin ligase FBXL14 which otherwise serves to target c-Myc for proteasomal degradation. And moreover, this was shown to be part of a regulatory feedback loop since NIPSNAP1 is subject to transcriptional repression by c-Myc. The second interaction involves NIPSNAP1 binding to superoxide dismutase 2 (SOD2) which enhances its association with SIRT3, thereby deacetylating SOD2 and increasing its activity, in turn, alleviating elevated ROS levels. Consequently, silencing NIPSNAP1 expression contributes to P27-mediated senescence via both decreasing the levels of c-Myc together with increasing ROS levels. Together these findings reveal an important role for NIPSNAP1 as a negative regulator of cancer cell senescence, which functions to sustain the viability of cancer cells under growth factor deprivation stress.

## Results

### NIPSNAP1 is upregulated following serum deprivation and promotes cell growth

Within the tumor microenvironment, cancer cells commonly experience states of nutritional deprivation and other insults largely arising from the combined effects of metabolic hyperactivity and limited circulation [35]. Consequently, cancer cells must mount adaptive responses to overcome deficiencies in nutritional components and growth factors, otherwise such conditions may compromise the proliferative capacity of cells, leading to cell cycle arrest [36]. In this study, we focused on determining the specific molecular mechanisms and/or signaling pathways invoked after serum deprivation in HCT116 cells, a representative cell line derived from human colon cancer. We hypothesized that proteins upregulated by serum deprivation were potential mediators of key adaptive responses. Accordingly, HCT116 cells were cultured with or without serum for 24 h before undertaking mass spectrometry (MS)-based proteomics to identify differentially expressed proteins (Additional file 1: Fig. S1A). We then selected the 15 most upregulated candidate proteins following serum removal for initial validation.

**Fig. 1:**
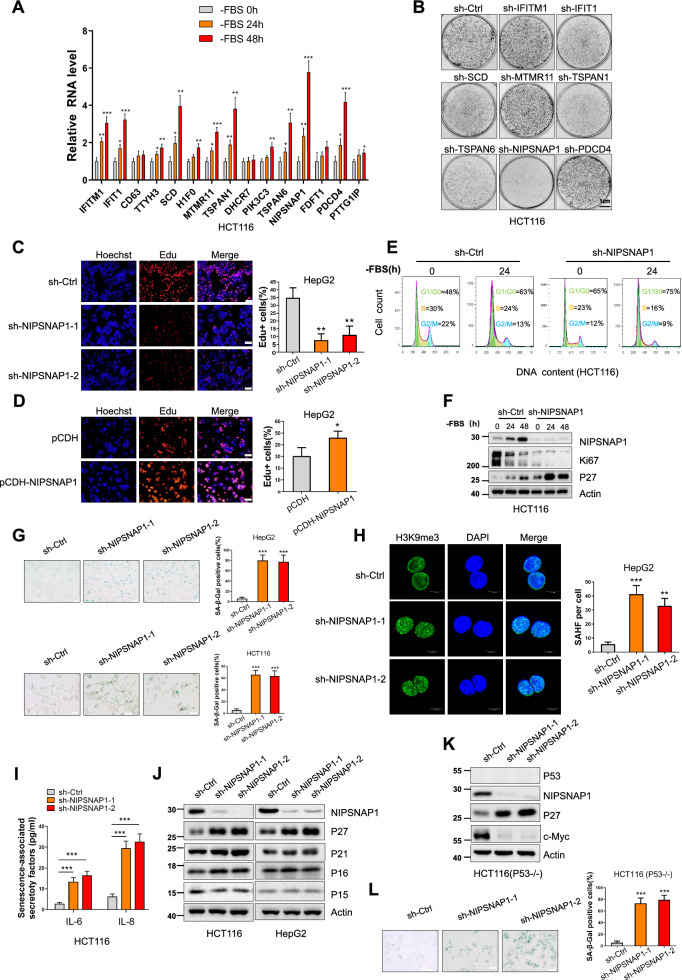
Screening identifies upregulation of NIPSNAP1 following serum deprivation promotes cell growth and inhibits cellular senescence. **A** HCT1116 cells were subjected to serum starvation for 0, 24 and 48 h before conducting qPCR measurements of 15 candidate proteins determined from MS analysis. **B** Clonogenic growth of HCT116 cells after 2 weeks of culture following lentiviral shRNA-mediated knockdown of selected candidate genes from **A** compared with an empty vector control (sh-Ctrl). **C**, **D** sh-Ctrl or two independent shRNAs targeting NIPSNAP1 (sh-NIPSNAP1-1 and sh-NIPSNAP1-2) or **D** after transduction with the pCDH empty vector or NIPSNAP1 overexpression vector pCDH-NIPSNAP1 was used to transduce HepG2 cells for 48 h, and DNA synthesis determined using EdU incorporation for 4 h in **C** HepG2 cells transduced with. Representative images showing nuclei (Hoechst staining, blue) or incorporated EdU (red) (left) were subjected to image analysis to determine comparative DNA synthesis rates (right) (bar = 100 µm). **E** Flow cytometric-based determination of cell cycle phases in HCT116 cells transduced with sh-Ctrl or sh-NIPSNAP1 cultured with or without serum (FBS) for 24 h. **F** Western blotting measuring the cellular levels of Ki67 and P27 in HCT116 cells treated with shRNAs as per **E**. Actin was used throughout as a loading control. **G** Detection of SA-β-gal staining in HCT116 and HepG2 cells transduced with sh-Ctrl or two independent-shRNAs targeting NIPSNAP1. Representative light micrographs (left) and the percentage of SA-β-gal-positive cells (right) (bar = 50 µm). **H** Senescence-associated heterochromatin foci (SAHFs) decorated by immunofluorescence (IF) staining against H3K9me3 (green) in HepG2 cells (left) transduced with sh-Ctrl or two independent-shRNAs targeting NIPSNAP1. DAPI counterstaining of nuclei is shown in blue (bar = 10 µm). Quantitation of SAHF/cell (right). **I** Senescence-associated secretory phenotype in HCT116 cells treated as per **G**. Secreted levels of IL-6 and IL-8 in culture supernatants were determined by ELISA. **J** Western blotting analysis of P27, P21, P16, and P15 levels in HCT116 and HepG2 cells after 48 h transduction with sh-Ctrl or two independent-shRNAs targeting NIPSNAP1. **K** HCT116 (P53−/−) cells were transduced with sh-Ctrl or two independent-shRNAs targeting NIPSNAP1 before analysis of P27 and c-Myc levels by Western blot. **L** SA-β-gal staining in HCT116 (P53−/−) cells as per **G** after NIPSNAP1 knockdown. **A**, **C**, **D**, **G**–**I** and **L** are mean ± SD; n = 3 independent experiments, two-tailed Student’s t test, Images in **B**, **E**, **F**, **J** and **K** represents three independent experiments (*P < 0.05; **P < 0.01, ***p < 0.001)

Quantitative polymerase chain reaction (qPCR) assays were employed to confirm changes in the corresponding mRNA levels of each gene. We observed that 12/15 candidate genes were significantly upregulated following sustained serum deprivation over 24–48 h (Fig. 1A). Among these candidates, eight genes whose mRNA levels were upregulated > 2.5 times were chosen for functional validation in shRNA-mediated knockdown studies in HCT116 cells. After individual gene silencing (Additional file 1: Fig. S1B), we assessed the impact on clonogenicity and cell growth using colony-forming and CCK-8 assays (Fig. 1B and Additional file 1: Fig. S1C). Notably, decreased proliferative phenotypes were associated with 4/8 genes and among these, NIPSNAP1 mRNA was most significantly increased in response to serum deprivation and its knockdown yielded the highest rate of growth inhibition compared to sh-control cells. On this basis we decided to focus on understanding how NIPSNAP1 contributes to the serum deprivation responses of cancer cells.

To ensure that the growth inhibitory phenotype resulted from NIPSNAP1 depletion and not off-target effects, we implemented two independent shRNAs targeting NIPSNAP1, namely sh-NIPSNAP1-1 and -1-2, respectively. Indeed, knockdown of NIPSNAP1 with either construct (Additional file 1: Fig. S1D) resulted in comparative reductions in cell proliferation as indicated by decreases in the rates of EdU incorporation indicative of cells entering S-phase (Fig. 1C). Conversely, ectopic expression of NIPSNAP1 (Additional file 1: Fig. S1E) increased the percentage of EdU-positive cells (Fig. 1D). Taken together, these results suggested that NIPSNAP1 functions to promote cancer cell proliferation while interfering with its expression appears to invoke cell cycle arrest.

### NIPSNAP1 inhibits cellular senescence through suppressing P27

To better define how NIPSNAP1 affects cell proliferation, particularly following serum withdrawal, we analyzed cell cycle parameters by flow cytometry. As expected, we found that serum deprivation resulted in increased numbers of cells in the G0/G1 phase with this phenomenon being exacerbated after knockdown of NIPSNAP1 (Fig. 1E). Cells arresting in G0/G1 phase are quiescent and commonly express lower levels of proliferation markers such as Ki67 while expressing higher levels of cell cycle inhibitors such as P27 [37, 38]. Consistently, immunoblotting analyses showed that removal of serum led to the downregulation of Ki67 and upregulation of P27, respectively, with these changes being far more pronounced after NIPSNAP1 knockdown (Fig. 1F), proposing that NIPSNAP1 acts to overcome the inhibitory effects of serum deprivation. However, the underlying mechanism whereby NIPSNAP1 affects cell cycle proliferation versus arrest remained to be determined.

Cell cycle arrest and the accompanying upregulation of P27 can be indicative of either temporary or permanent cell state changes involving quiescence and senescence, respectively [39, 40]. To examine whether senescence programming was activated upon NIPSNAP1 depletion, we first conducted assays for senescence-associated (SA) β-gal activity together with staining for H3K9me3 which marks the accumulation of senescence-associated heterochromatin foci (SAHF). Notably, we found significant increases in the proportion of SA-β-gal-positive cells following the knockdown of NIPSNAP1 with a striking ~ 80% of total cells being positive (Fig. 1G). Moreover, this phenotype was evident in both the HCT116 and HepG2 cell lines, indicative that the results were not restricted to a single cell line. Consistently, NIPSNAP1 knockdown produced increases in the density of SAHF in HepG2 cells (Fig. 1H). Further assessment of inflammatory cytokine secretion showed that both IL-6 and IL-8 levels were increased following NIPSNAP1 knockdown (Fig. 1I), indicative of the SASP [2]. Collectively, these results assays established that the major phenotype associated with the knockdown of NIPSNAP1 involved cellular senescence.

We further extended our immunoblotting analyses to determine whether other cell cycle inhibitors associated with senescence were involved including P21, P16 and P15. However, knockdown of NIPSNAP1 most strongly induced increases in P27 and to a lesser extent, P21 (Fig. 1J). As the activation of the P21 signaling network involves P53 whereas P27 signaling does not [41, 42], we examined the actions of NIPSNAP1 in HCT116 cells lacking P53 expression (P53−/−). Indeed, we found that NIPSNAP1 knockdown in the absence of P53 still caused the robust upregulation of P27 although intriguingly, there was an obvious downregulation of c-Myc levels (Fig. 1K). As anticipated, silencing of NIPSNAP1 in HCT116 (P53−/−) cells showed similar increases in the proportion of SA-β-gal positive cells as observed in wildtype P53-expressing HCT116 cells (Figs. 1L). Thus, cellular senescence induced by knockdown of NIPSNAP1 appears to be independent of the P53 pathway.

### c-Myc transcriptionally represses NIPSNAP1

**Fig. 2:**
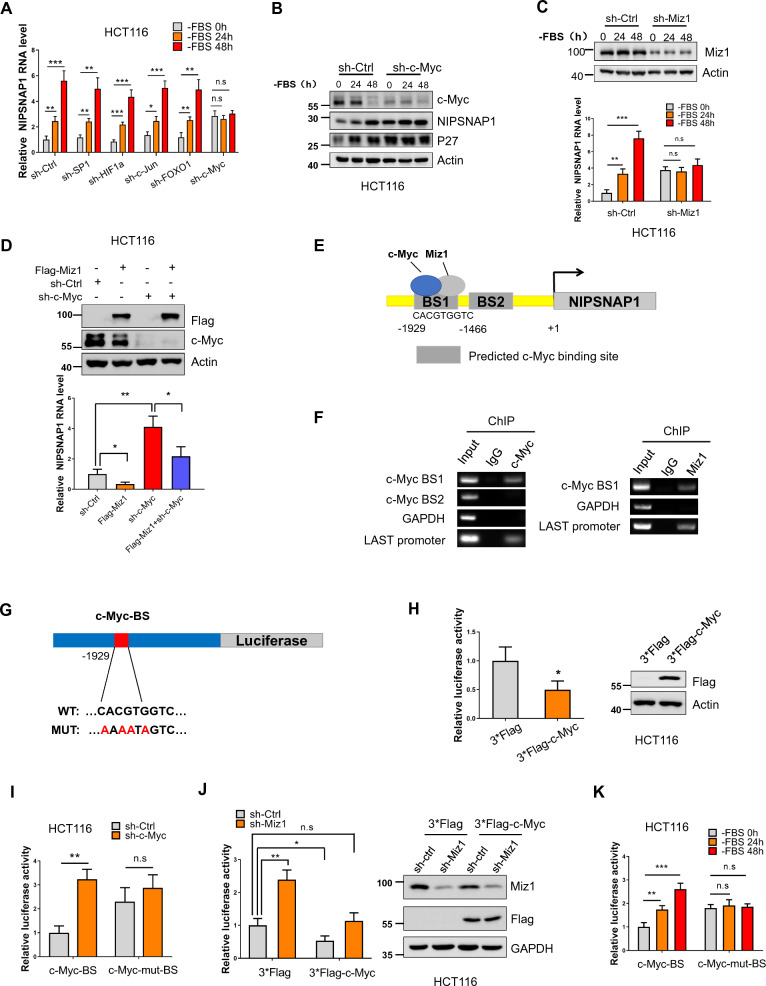
NIPSNAP1 expression is transcriptionally repressed by c-Myc-Miz1 with de-repression in response to serum deprivation. **A** Relative NIPSNAP1 mRNA levels in HCT116 cells after transduction with sh-Ctrl, sh-SP1, sh-HIF1a, sh-c-Jun, sh-FOXO1 or sh-c-Myc lentiviruses determined by qPCR at 0, 24 and 48 h following FBS withdrawal. **B** Western blotting measurements of NIPSNAP1 and P27 levels in HCT116 cells treated with sh-Ctrl or sh-c-Myc lentiviruses at 0, 24 and 48 h following FBS withdrawal. Actin was used throughout as a loading control. **C** HCT116 cells were transduced with sh-Ctrl or sh-Miz1 lentiviruses at 0, 24 and 48 h following serum (FBS) withdrawal. Cell lysates were subjected to Western blotting against Miz1 or actin (top) or total RNA used for qPCR-based measurement of NIPSNAP1 mRNA (bottom). **D** HCT116 cells were transduced with sh-Ctrl or sh-c-Myc in combination with empty Flag-vector or the Flag-Miz1 overexpression vector. Cell lysates were subjected to Western blotting against Flag or c-Myc (top) or total RNA used for qPCR-based measurement of NIPSNAP1 mRNA (bottom). **E** Schematic illustrating the putative c-Myc binding sites (BSs) in the NIPSNAP1 promoter, termed c-Myc-BS1 (− 1929 to − 1921 bp) and c-Myc-BS2 (− 279 to − 272 bp), respectively. **F** ChIP assays targeting c-Myc (left) or Miz1 (right) and the corresponding control IgG samples performed in HCT116 cells. Primers targeting c-Myc-BS1 and c-Myc-BS2 were used along with negative and positive control primers against the GAPDH and LAST promoters, respectively, the latter a known target of c-Myc/Miz1. **G** Schematic illustrating the design of pGL3-based luciferase reporter constructs containing the wildtype (WT) or mutant c-Myc binding sites in the NIPSNAP1 promoter. **H** Luciferase reporter assays conducted in HCT116 cells measuring the activity of the WT NIPSNAP1 reporter construct after co-transfection of the empty 3*Flag-vector or 3*Flag-c-Myc overexpression vector. Relative luciferase activity (left) and Western blotting to confirm ectopic c-Myc expression. **I** Luciferase reporter assays were conducted as per **H** after transduction with sh-Ctrl or sh-c-Myc and co-transfection with either the wildtype (WT) or mutant (Mut) c-Myc reporter constructs. **J** Luciferase reporter assays were conducted as per **H** after transduction with sh-Ctrl or sh-Miz1 and co-transfection with the empty 3*Flag-vector or 3*Flag-c-Myc overexpression vector. Relative luciferase activity (left) and Western blotting to confirm Miz1 knockdown and ectopic c-Myc expression. **K** Luciferase reporter assays were conducted as per **H** after transduction with either the wildtype (WT) or mutant (Mut) c-Myc reporter constructs at 0, 24 and 48 h following serum (FBS) withdrawal. **A**, **C**, **D** and **H**–**K** are mean ± SD; n = 3 independent experiments, two-tailed Student’s t test, Images in **B** and **F** represents three independent experiments (*P < 0.05; **P < 0.01, ***p < 0.001)

We next sought to determine the upstream mechanism underlying the induction of NIPSNAP1 following serum deficiency. Given the patent increase in NIPSNAP1 transcript levels we first considered whether this resulted from the increased transcription and/or decreased degradation of NIPSNAP1 mRNA. Using the JASPAR database [43] to predict conserved transcription factor (TF) binding sites within the proximal promoter region of *NIPSNAP1* revealed high scoring matches with several stress-associated TFs including SP1, c-Myc, HIF1a, FOXO1 and c-Jun (Additional file 2: Fig. S2A). To test their respective involvement, each TF was individually knocked down in combination with serum deprivation treatment for 24 h and 48 h, and the levels of NIPSNAP1 was examined. Surprisingly, silencing c-Myc prevented the upregulation of NIPSNAP1 mRNA in response to serum withdrawal while knockdown of the other TFs had no significant effect (Fig. 2A). Moreover, knockdown of c-Myc resulted in increased levels of NIPSNAP1 mRNA relative to the shRNA control levels with similar findings evident at the protein level (Fig. 2B and Additional file 2: Fig. S2B). Together this suggested that c-Myc normally transcriptionally inhibits NIPSNAP1 expression.

Since the cofactor Miz1 prominently cooperates with c-Myc in the transcriptional repression of target genes [44, 45], we examined the role of Miz1 in the regulation of NIPSNAP1 during the serum deprivation response in HCT116 cells. Indeed, we observed that silencing of endogenous Miz1 increased NIPSNAP1 mRNA levels under basal conditions while also completely ablating the transcriptional increases elicited by serum withdrawal (Fig. 2C). Conversely, we determined that Miz1 overexpression dampened basal NIPSNAP1 mRNA levels while also attenuating the upregulation of NIPSNAP1 associated with c-Myc knockdown (Fig. 2D). These findings indicated that Miz1 cooperates with c-Myc to transcriptionally repress NIPSNAP1. To further strengthen this conclusion, we conducted chromatin immunoprecipitation (ChIP) assays. Noting two predicted c-Myc/MIZ1 binding sites (BSs) located in the NIPSNAP1 promoter (Fig. 2E), ChIP assays indicated that c-Myc-BS1 (− 1929 to − 1921 bp) but not c-Myc-BS2 (− 279 to − 272 bp) was recovered with c-Myc antibodies, with parallel assays confirming that Miz1 co-occupies the c-Myc-BS1 site with c-Myc (Fig. 2F). Furthermore, we constructed luciferase reporter vectors containing the NIPSNAP1 promoter sequence with either intact or mutated c-Myc-BS1 sequences (Fig. 2G). Measuring luciferase levels after transfecting these vectors into HCT116 cells revealed that transcriptional activity of the wildtype NIPSNAP1 construct was diminished when expressed in combination with c-Myc (Fig. 2H). In contrast, knockdown of c-Myc produced increases in the transcriptional activity of the wildtype but not the mutant NIPSNAP1 vector (Fig. 2I). Supporting the role of the c-Myc/Miz1 interaction in regulating NIPSNAP1 levels, knockdown of Miz1 enhanced the transcriptional reporter activity of the NIPSNAP1 promoter construct while overexpression of c-Myc significantly suppressed this effect (Fig. 2J). Finally, examination of the transcriptional responses of the NIPSNAP1 reporter vectors following serum deprivation showed that the wildtype but not the mutant construct responded to serum withdrawal (Fig. 2K). Together these data definitively demonstrate that c-Myc-Miz1 transcriptionally inhibit NIPSNAP1 under basal conditions with serum deprivation serving to lift this repression.

### NIPSNAP1 affects the stability of the c-Myc protein

**Fig. 3:**
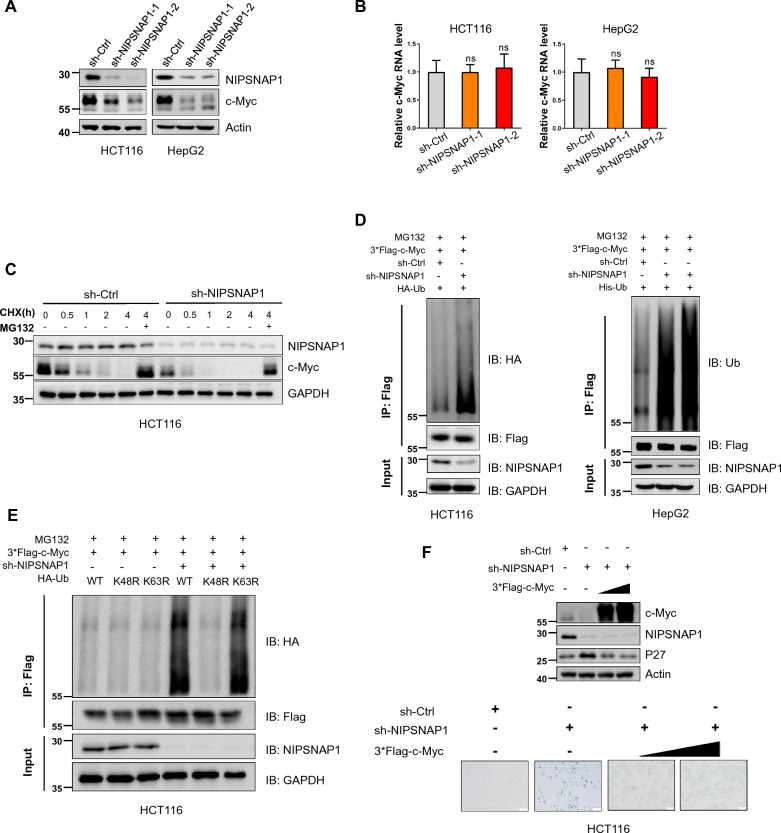
NIPSNAP1 promotes c-Myc protein stability as part of a mutual regulatory feedback loop. **A** Western blotting analysis of c-Myc levels in HCT116 and HepG2 cells after transduction with sh-Ctrl or two independent-shRNAs targeting sh-NIPSNAP1. Actin was used as a loading control throughout. **B** Parallel assays conducted in the cells from **A** measuring c-Myc mRNA levels using qPCR. **C** Cycloheximide (CHX) chase assays comparing the stability of c-Myc in HCT116 cells after transduction with sh-Ctrl or sh-NIPSNAP1 lentiviruses. The cells were pretreated with 50 mg/ml CHX for 0–4 h before analysis of NIPSNAP1 and c-Myc expression levels by Western blotting. As indicated, the effects of co-treating cells with or without 10 mM MG132 were determined at the 4 h timepoint. **D** Analysis of the polybiquitylation of ectopically expressed c-Myc. HCT116 (left) or HepG2 (right) cells were transduced with sh-Ctrl or sh-NIPSNAP1 before co-transfection with hemagglutinin (HA)-Ub and 3*Flag-c-Myc. Cell lysates (input) were subjected to Western blotting against NIPSNAP1 and a GAPDH loading control whereas anti-Flag immunoprecipitates (IPs) were blotted for anti-Ub or anti-HA as shown. **E** Ubiquitination analysis of 3*Flag-c-Myc measured as per **D** in HCT116 cells co-transfected without or with sh-NIPSNAP1 in combination with either HA-Ub WT, HA-Ub K48R, or HA-Ub K63R, respectively. **F** HCT116 cells transduced with sh-Ctrl or sh-NIPSNAP1 in combination with two different concentrations of 3*Flag-c-Myc were subjected to Western blotting to measure c-Myc, NIPSNAP1 and P27 (top) or SA-β-gal staining (bottom), respectively. **B** is mean ± SD; n = 3 independent experiments, two-tailed Student’s t test, Images in **A** and **C**–**F** represents three independent experiments (*P < 0.05; **P < 0.01, ***p < 0.001)

Prior reports have shown that the inactivation of c-Myc promotes cancer cell senescence [12, 46] with c-Myc also exerting a significant suppressive effect on P27 expression [47, 48]. Similarly, we found that senescence induction following NIPSNAP1 depletion involved P27, raising the possibility that NIPSNAP1 may also affect the expression of c-Myc. Consequently, we examined the impact of NIPSNAP1 knockdown on the levels of c-Myc. Instructively, we observed that the levels of c-Myc were significantly downregulated in both in HCT116 and HepG2 cells following NIPSNAP1 depletion (Fig. 3A), prompting us to further explore the mechanism underlying the actions of NIPSNAP1 on c-Myc expression.

First, we examined if the decreases in c-Myc protein resulted from changes at the transcriptional level. However, knockdown of NIPSNAP1 failed to affect c-Myc mRNA (Fig. 3B), suggesting that NIPSNAP1 exerts post-transcriptional effects on c-Myc. To assess the stability of c-Myc protein we implemented cycloheximide (CHX) chase assays, showing that the half-life of c-Myc was reduced following NIPSNAP1 knockdown (Fig. 3C). Moreover, the addition of MG132 in these assays served to rescue the diminished levels of c-Myc, indicative that NIPSNAP1 functions to maintain the stability of c-Myc protein by preventing its proteasomal-mediated turnover. Supporting this notion, c-Myc polyubiquitylation was increased following NIPSNAP1 knockdown in both HCT116 and HepG2 cell lines (Fig. 3D). To further determine whether c-Myc polyubiquitylation occurred on either lysine (K)48 or K63-linked ubiquitin chains, we implemented co-transfection assays using the Ub-K48R and Ub-K63R ubiquitin mutants. Notably, we observed that co-expression of Ub-K63R but not Ub-K48R in combination with NIPSNAP1 knockdown greatly increased the ubiquitylation of c-Myc (Fig. 3E), consistent with the notion that NIPSNAP1 prevents Lys48-linked ubiquitylation, the main targeting signal for degradation by the proteasome [49].

To lastly reconcile the relationship between senescence induction, NIPSNAP1 and c-Myc, we assessed whether ectopic expression of c-Myc could rescue the senescence phenotype evident following NIPSNAP1 knockdown. As anticipated, knockdown of NIPSNAP1 promoted increases in P27 levels with accompanying increases in cell senescence measured as SA-β-gal activity; however, ectopic c-Myc expression in combination with NIPSNAP1 knockdown largely prevented the increases in P27 levels and significantly reversed the induction of cell senescence caused by NIPSNAP1 knockdown (Fig. 3F). Together these data establish that NIPSNAP1 plays an important role in cancer cell homeostasis via stabilization of the c-Myc protein, with a negative feedback loop formed between c-Myc and NIPSNAP1 acting to modulate their respective expression.

### Competition between NIPSNAP1 and the E3 ligase FBXL14 dictates the stability of c-Myc protein

**Fig. 4:**
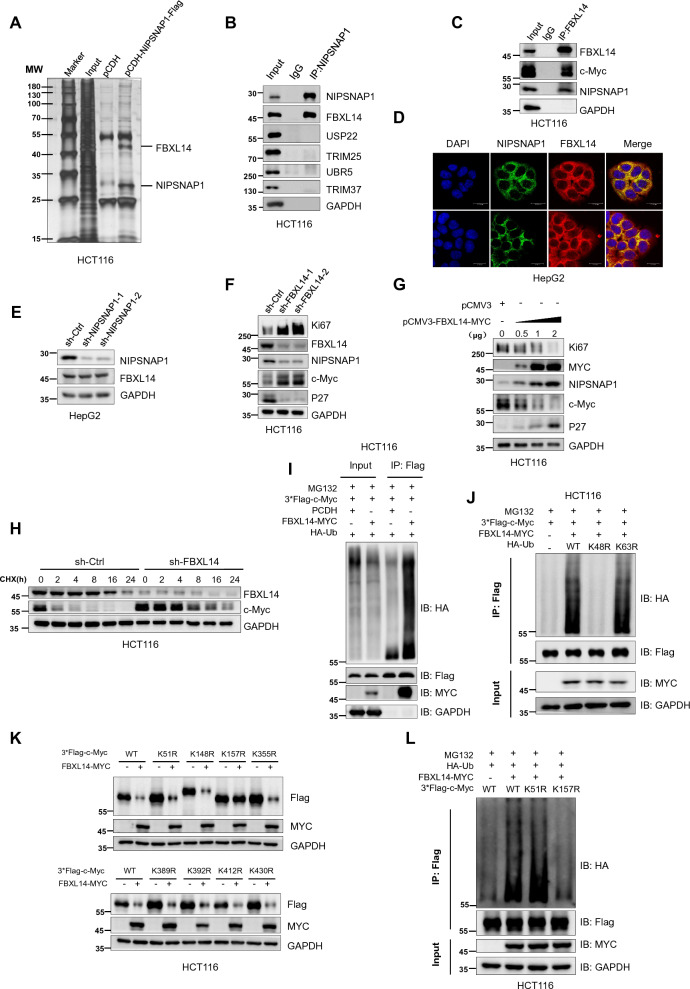
NIPSNAP1 sequesters FBXL14 to inhibit c-Myc degradation. **A** Fast Silver Staining gel comparing anti-Flag immunoprecipitants from HCT116 cells transduced with either a control vector (pCDH) or Flag-tagged NIPSNAP1 construct (pCDH-NIPSNAP1-Flag). The highlighted bands denoted as FBXL14 and NIPSNAP1, respectively, were assigned based on mass spectrometry (MS/MS) analysis tabulated elsewhere as Additional file 8: Table S6. **B** Western blotting-based assessment of interactions between endogenous NIPSNAP1 and candidate ubiquitin modifiers detected in **A** and from the literature associated with c-Myc regulation. Only FBXL14 was recovered within NIPSNAP1 immunoprecipitates (IPs) from HCT116 cells. GAPDH was used as a loading control throughout. **C** Endogenous NIPSNAP1 is recovered within FBXL14 IPs from HCT116 cells conducted as per **B**. **D** Representative confocal images showing immunofluorescence staining against NIPSNAP1 (green), FBXL14 (red), and DAPI (blue) in HepG2 cells (bar = 20 µm). **E** Western blotting analysis of FBXL14 levels in HepG2 cells after transduction with sh-Ctrl or two independent-shRNAs targeting NIPSNAP1. **F** Western blotting analysis of the levels of Ki67, NIPSNAP1, c-Myc and P27 in HCT116 cells after transduction with sh-Ctrl or two independent-shRNAs targeting FBXL14. **G** Western blot analysis of HCT116 cells as per **F** after transduction with graded concentrations of FBXL14-MYC. Ectopic FBXL14 levels were detected with anti-Myc epitope antibodies. **H** Cycloheximide (CHX) chase assays comparing the stability of c-Myc in HCT116 cells after transduction with sh-Ctrl or sh-FBXL14 lentiviruses. The cells were pretreated with 50 mg/ml CHX for 0–24 h before analysis of FBXL14 and c-Myc expression levels by Western blotting. **I** Analysis of the effects of FBXL14 on the polybiquitylation of c-Myc. HCT116 cells were transduced with FBXL14-MYC and co-transfected with hemagglutinin (HA)-Ub and 3*Flag-c-Myc. Input samples or anti-Flag IPs were then subjected to Western blotting against HA, Flag and MYC, respectively. **J** Ubiquitination of 3*Flag-c-Myc measured as per **I** in cells co-transfected with FBXL14-MYC in combination with either HA-Ub WT, HA-Ub K48R, or HA-Ub K63R, respectively. **K** The stability of the indicated Flag-tagged lysine substitution mutants of c-Myc in the absence (empty vector) and presence of ectopically expressed FBXL14-MYC was measured in HCT116 cells. The expression of c-Myc and FBXL14 was revealed by Western blotting using antibodies against Flag and MYC, respectively. **L** Ubiquitylation assays performed in HCT116 cells after individually transfecting WT Flag-tagged c-Myc, or the indicated substitution mutants together with HA-Ub and FBXL14-MYC. Cell lysates (input) were subjected to Western blotting against MYC and a GAPDH loading control whereas anti-Flag immunoprecipitates (IPs) were blotted with anti-Flag and anti-HA, respectively

The preceding experiments revealed that NIPSNAP1 enhances the stability of the c-Myc protein via preventing its proteasomal destruction. To better understand the precise mechanisms involved, we hypothesized that interrogating proteins interacting with NIPSNAP1 would provide an essential clue. Towards this, we overexpressed epitope tagged (Flag) NIPSNAP1 in HCT116 cells and immunoprecipitated NIPSNAP1 before subjecting the samples to SDS-PAGE. Silver staining of the gel showed two conspicuous bands selectively precipitated from cells transfected with NIPSNAP1-Flag but not the control vector (Fig. 4A). Mass spectrometry was then used to reveal the potential identities of these proteins with the lower band identified as NIPSNAP1 while the upper band included the E3 ligases FBXL14, TRIM37 and UBR5 (Additional file 8: Table S6). Instructively, blotting analysis of the immunoprecipitated samples for each of the candidate E3 ligases revealed that FBXL14 [50] was coprecipitated with NIPSNAP1 while TRIM37 and UBR5 were absent along with USP22 and TRIM25 that were included as controls (Fig. 4B). Moreover, further analyses indicated that NIPSNAP1 could be reciprocally immunoprecipitated with FBXL14 (Fig. 4C), indicative of a robust interaction between NIPSNAP1 and FBXL14. Consistent with this result, confocal imaging revealed there was significant colocalization between NIPSNAP1 and FBXL14 in the cytoplasmic space of HepG2 cells (Fig. 4D).

We next investigated the functional significance of the NIPSNAP1-FBXL14 interaction. Importantly, knockdown of NIPSNAP1 had no impact on the protein levels of FBXL14 (Fig. 4E), suggesting that their interaction did not relate to the stability of FBXL14. An alternate possibility was that FBXL14 catalyzes the polyubiquitylation of NIPSNAP1 that would be expected to direct its proteasomal turnover. Nonetheless, knockdown of FBXL14 yielded the surprising result showing that NIPSNAP1 levels were diminished (not increased) which accompanied increases in the expression of Ki67 and c-Myc while there was a downregulation of P27 (Fig. 4F). Conversely, the introduction of ectopic Flag-tagged FBXL14 increased the levels of NIPSNAP1 and P27 while decreasing the expression of Ki67 and c-Myc (Fig. 4G). Focusing on whether FBXL14 affects the stability of c-Myc, we found that knockdown of FBXL14 significantly prolonged the half-life of c-Myc (Fig. 4H) whereas the overexpression of FBXL14 was associated with increases in the polyubiquitylation of c-Myc (Fig. 4I). Moreover, we further established that FBXL14 promotes the polyubiquitylation of c-Myc through Lys48-linkages (Fig. 4J), much like our analysis of c-Myc ubiquitylation following knockdown of NIPSNAP1. Together this data suggested that c-Myc is an E3 ubiquitin ligase substrate of FBXL14.

To further identify the ubiquitylation site(s) in c-Myc targeted by FBXL14, we interrogated the PhosphoSitePlus [51] and hCKSAAP_UbSite [52] databases. Lysine residues in c-Myc providing the highest predictive scores were subjected to site-directed mutagenesis to create lysine to arginine mutants involving K51R, K148R, K157R, K355R, K389R, K392R, K412R, and K430R. Thereafter, implementing the transfection of these mutants in combination with the overexpression of FBXL14 showed that only co-transfection with the c-Myc-K157R mutant resulted in the stabilization of c-Myc in the presence of c-Myc-K157R (Fig. 4K). Consistently, the decreased polyubiquitylation signals in wildtype c-Myc resulting from FBXL14 overexpression were phenocopied with c-Myc-K157R but not c-Myc-K51R (Fig. 4L). Together these results indicated that FBXL14 catalyzes the ubiquitylation of lysine 157 in c-Myc, which was further confirmed using mass spectrometry (Additional file 9: Table S7). Nevertheless, the nature of the relationship between FBXL14 and NIPSNAP1 remained an outstanding question. However, analysis of the different c-Myc mutants after NIPSNAP1 knockdown provided an important clue, namely, only the levels of the K157R mutant remained stable (Additional file 2: Fig. S2C), which was associated with the blockade of polyubiquitylation (Additional file 2: Fig. S2D).

**Fig. 5:**
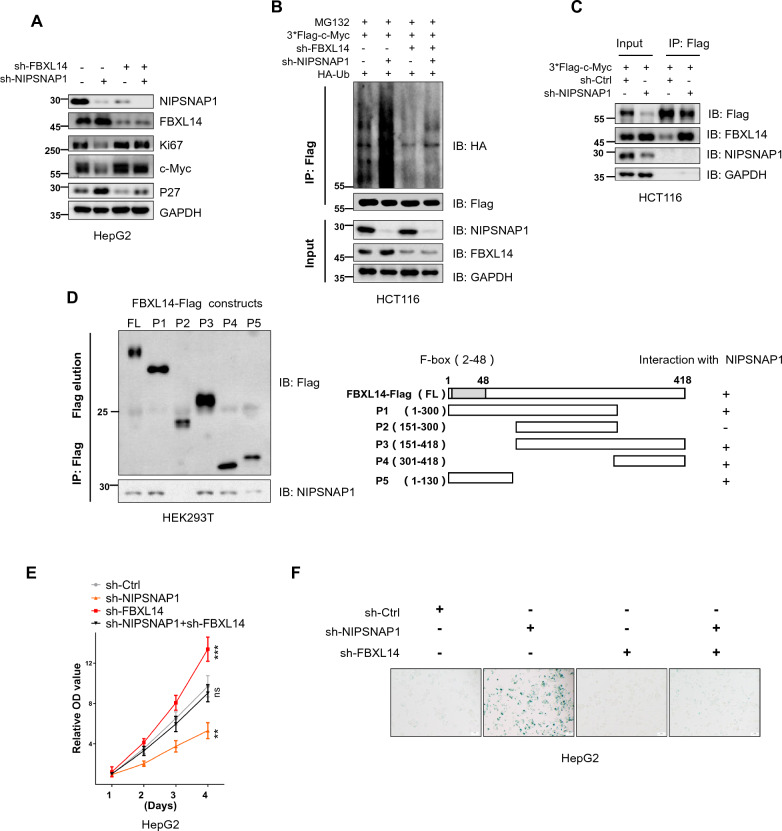
Dissecting interactions between NIPSNAP1, FBXL14 and c-Myc. **A** Western blotting analysis of the levels of NIPSNAP1, FBXL14, Ki67, c-Myc, and P27 in HepG2 cells subjected to transduction with sh-Ctrl (−) or sh-NIPSNAP1 alone or in combination with sh-FBXL14. GAPDH was used a loading control throughout. **B** Ubiquitylation assays performed in HCT116 cells transduced with sh-Ctrl, sh-FBXL14 or sh-NIPSNAP1 in combination with co-transfection with hemagglutinin (HA)-Ub and 3*Flag-c-Myc. Cell lysates (input) were subjected to Western blotting against NIPSNAP1 and FBXL14 whereas anti-Flag immunoprecipitates (IPs) were blotted with anti-Flag and anti-HA, respectively. **C** HCT116 cells were transduced with 3*Flag-c-Myc in combination with sh-Ctrl or sh-NIPSNAP1 before Western blotting analysis of cell lysates or anti-Flag immunoprecipitates against Flag, FBXL14 and NIPSNAP1, respectively. **D** Schematic illustrating the design of full-length Flag-tagged FBXL14 (FBXL14-WT) and overlapping truncated constructs (FBXL14-P1, -P2, -P3, P4 and P5, respectively; left). HCT116 cells were transfected with the indicated constructs and subjected to immunoprecipitation with anti-Flag antibodies before Western blotting against Flag and NIPSNAP1, respectively (right). **E**, **F** HepG2 cells were subjected to transduction with sh-Ctrl (−) or sh-NIPSNAP1 alone or in combination with sh-FBXL14 before assessment of cell proliferation using CCK-8 assays (**E**) and cellular senescence as SA-β-gal staining (**F**). **E** Is mean ± SD; n = 3 independent experiments, two-tailed Student’s t test, images in **A**–**D** and **F** represents three independent experiments (*P < 0.05; **P < 0.01, ***p < 0.001)

Expanding on this point, we found that knockdown of FBXL14 reversed the downregulation of c-Myc resulting from the silencing of NIPSNAP1 (Fig. 5A). Moreover, co-knockdown of NIPSNAP1 and FBXL14 decreased ubiquitylation of c-Myc (Fig. 5B), implying that NIPSNAP1 enhances the stability of c-Myc by binding to and sequestering FBXL14. Consistent with this notion, the comparative levels of interaction between FBXL14 and c-Myc were enhanced after knockdown of NIPSNAP1 (Fig. 5C). To further dissect which region of NIPSNAP1 binds to FBXL14, we constructed five overlapping truncation constructs of FBXL14 designated P1-P5 divided according to length. After individually co-transfecting each of these constructs along with NIPSNAP1 into HEK293T cells, we observed binding between and NIPSNAP1 and full length FBXL14 and all truncated constructs except for P2 (Fig. 5D), indicative that the FBXL14-NIPSNAP1 interaction occurs in both the amino terminal (P5) and carboxyl terminal (P4) sequences in FBXL14.

Lastly, we sought to validate that the growth arrest and cell senescence phenotypes accompanying NIPSNAP1 knockdown resulted from the contribution of FBXL14 and NIPSNAP1 to the homeostasis of c-Myc levels. Indeed, the trajectory of expression changes in c-Myc, Ki67 and P27 following either knockdown or overexpression of FBXL14 (Fig. 4F, G) suggested that NIPSNAP1 and FBXL14 functioned in a competitive manner to exert regulation over c-Myc levels. Consequently, we compared the phenotypic effects of NIPSNAP1 knockdown alone and in combination with FBXL14. As might be anticipated, knockdown of FBXL14 increased the proliferative capacity of cells and moreover, the diminished proliferation rates accompany NIPSNAP1 knockdown were rescued in co-knockdown cells (Fig. 5E). Moreover, the high levels of cell senescence following knockdown of NIPSNAP1 were similarly reverted to by co-knockdown of FBXL14 with NIPSNAP1 (Fig. 5F). Collectively, our results propose that the NIPSNAP1 FBXL14 interaction serves to compete out the interaction between FBXL14 and c-Myc, which as a result, prevents the ubiquitylation and proteasomal degradation of c-Myc. In turn, disrupting this balance by manipulating NIPSNAP can induce cellular senescence through the downregulation of c-Myc and consequent upregulation of P27.

### NIPSNAP1 can inhibit ROS-induced cellular senescence in a SOD2 dependent manner

Based on the preceding findings, we sought to define how cellular senescence was induced by NIPSNAP1 knockdown. Noting previous reports linking the induction of cellular senescence with imbalances in cell redox [53–55], and in particular the upregulation of P27 levels accompanying ROS accumulation [56, 57], we investigated the relationship between NIPSNAP1 expression and cellular ROS levels.

**Fig. 6:**
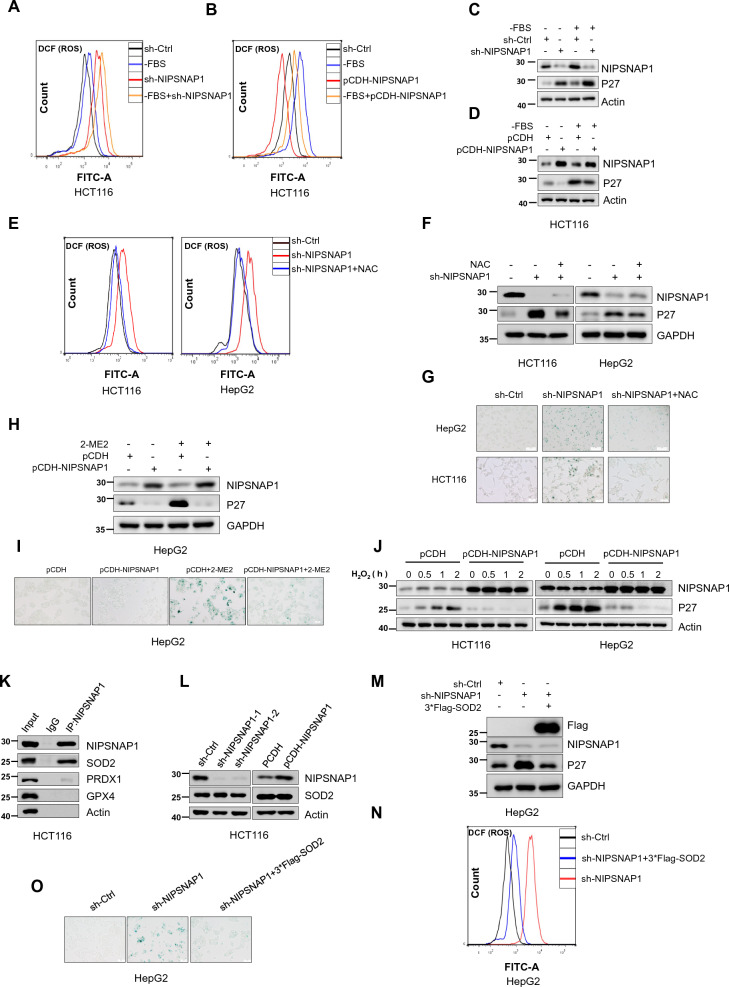
NIPSNAP1 inhibits ROS-induced cellular senescence in a SOD2-dependent manner. **A**–**D** HCT116 cells transduced with sh-Ctrl or sh-NIPSNAP1 or with pCDH or the pCDH-NIPSNAP1 overexpression vector were cultured with or without FBS for 24 h. ROS levels were determined using the DCF probe by flow cytometry (**A**, **B**). Cell lysates were subjected to Western blotting to measure NIPSNAP1 and P27 (**C**, **D**). **E** HCT116 and HepG2 cells transduced with sh-Ctrl or sh-NIPSNAP1 were cultured with or without the ROS scavenger NAC (4 mM) for 6 h. ROS levels were determined using the DCF probe by flow cytometry. **F**, **G** The cells from **E** were subject to parallel Western blotting analysis against NIPSNAP1, P27 and the GAPDH loading control (**F**) or subjected to SA-β-gal staining analysis (**G**), respectively. **H**, **I** HepG2 cells were transduced with pCDH or pCDH-NIPSNAP1 and cultured with or without the ROS activator 2-ME2 (10 mM) for 24 h. Cell lysates were subjected to Western blotting to measure NIPSNAP1 and P27 (**H**) or the cells subjected to SA-β-gal staining analysis (**I**). **J** HCT116 and HepG2 cells transduced with pCDH or pCDH-NIPSNAP1 were treated with 0.5 mM H_2_O_2_ for 0–2 h before analysis of NIPSNAP1 and P27 expression levels by Western blot. Actin was used as a loading control throughout. **K** Immunoprecipitation (IP) analyses against endogenous NIPSNAP1 undertaken in HCT116 cells examining interactions with SOD2, PRDX1 and GPX4. **L** Western blotting analysis of SOD2 levels in HCT116 cells after transduction with sh-Ctrl or two independent-shRNAs targeting (sh-NIPSNAP1-1 and -2, left), or after transduction with pCDH or the pCDH-NIPSNAP1 overexpression vector (right). **M**–**O** HepG2 cells were transduced with sh-Ctrl or sh-NIPSNAP1 alone or in combination with 3*Flag-SOD2. Thereafter, the cells were subjected to Western blot analysis against Flag, NIPSNAP1 and P27 (**M**), the detection of ROS levels by flow cytometry using the DCF probe (**N**), and SA-β-gal staining (**O**) analysis, respectively. **A**–**O** All data are representative of three independent experiments

Instructively, we observed that serum deprivation increased cellular ROS levels in HCT116 cells while NIPSNAP1 knockdown exacerbated such increases (Fig. 6A). Conversely, the overexpression of NIPSNAP1 reduced basal ROS levels while blunting the increased ROS levels resulting from serum removal (Fig. 6B). Moreover, the relative expression levels of P27 in cells were concordant with the total ROS levels measured (Fig. 6C, D). Further investigations showed that increases in cellular ROS levels resulting from knockdown of NIPSNAP1 were alleviated through the addition of the ROS scavenger *N*-acetyl cysteine (NAC) (Fig. 6E). Moreover, NAC also partially reversed the increased levels of P27 associated with NIPSNAP1 knockdown which also diminished the induction of senescence as shown in parallel SA-β-gal staining assays (Fig. 6F, G). In contrast, the addition of the ROS activator 2-methoxyestradiol (2-ME2) resulted in P27 upregulation and accompanying senescence induction which were substantially reversed by the ectopic expression of NIPSNAP1 (Fig. 6H, I, respectively). Alternatively, we evaluated the effects of H_2_O_2_, a commonly used treatment that rapidly increases cellular ROS levels and triggers senescence [58, 59]. Notably, H_2_O_2_ induced time dependent increases in P27 expression while the overexpression of NIPSNAP1 substantially prevented the induction of P27 (Fig. 6J). Taken together, these results suggest that the antagonistic actions of NIPSNAP1 in senescence induction involve relieving the accumulation of ROS.

Next, we investigated the molecular mechanism through which NIPSNAP1 inhibits ROS. The primary antioxidant systems that control ROS levels include the superoxide dismutases (SODs), which convert O_2_ to H_2_O_2_, and the enzymes that convert H_2_O_2_ to water, including peroxidases (PRDXs) and glutathione peroxidases (GPXs) [60]. Thus, we hypothesized that the increased ROS induced by knockdown of NIPSNAP1 might be due to effects on one or more oxidoreductase systems. Of relevance, we found that SOD2 coprecipitated with NIPSNAP1 in our MS-based screen (Additional file 8: Table S6) and we further validated that SOD2 but not the representative PRDX member, peroxiredoxin 1 (PRDX1), or GPX member, glutathione peroxidase 4 (GPX4), bound to NIPSNAP1 (Fig. 6K). However, we found there were no significant changes in SOD2 protein levels following the knockdown or overexpression of NIPSNAP1 in HCT116 cells (Fig. 6L). Nevertheless, overexpressing SOD2 in cells which had been subjected to knockdown of NIPSNAP1 was sufficient to reverse the increases in P27 and ROS levels together with preventing the induction of senescence (Fig. 6M–O, respectively). Collectively this suggests that SOD2 plays a critical role in the inhibition of intracellular ROS by NIPSNAP1.

### NIPSNAP1 increases the antioxidant activity of SOD2 through SIRT3-mediated deacetylation

**Fig. 7:**
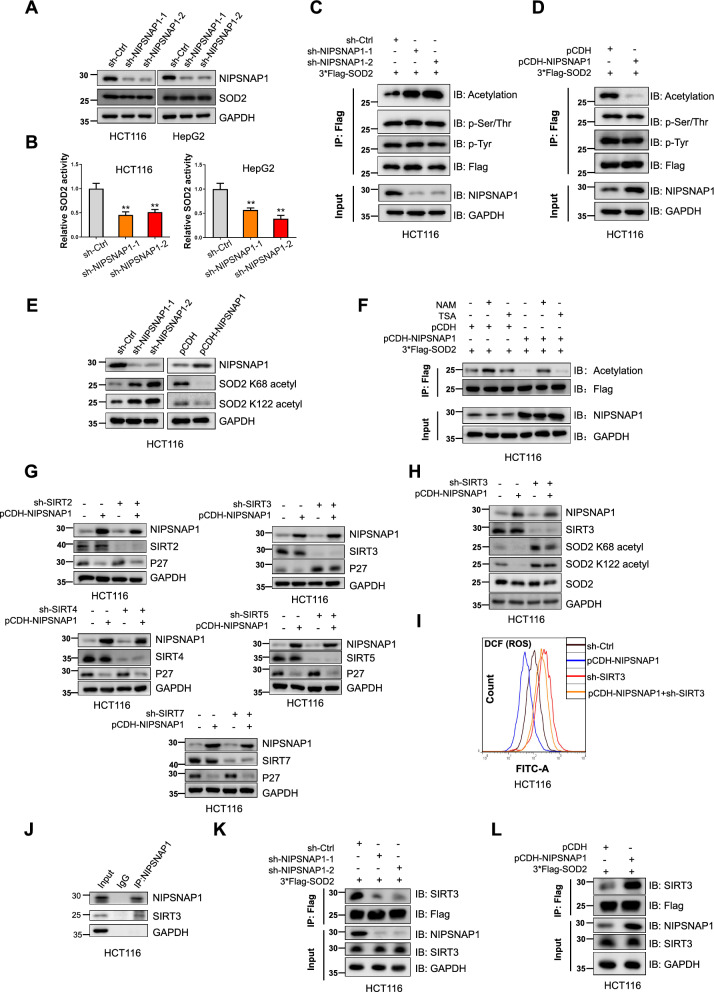
NIPSNAP1 increases the antioxidant activity of SOD2 through SIRT3-mediated deacetylation. **A**, **B** HCT116 (left) and HepG2 (right) cells transduced with sh-Ctrl or two independent-shRNAs targeting (sh-NIPSNAP1-1 and -2, respectively) were subjected to Western blotting to measure NIPSNAP1 and SOD2 levels (**A**) or manganese superoxide dismutase (MnSOD) activity (**B**), respectively. **C**, **D** HCT116 cells transduced with sh-Ctrl or two independent-shRNAs targeting (sh-NIPSNAP1-1 and -2, respectively) (**C**) or with pCDH or the pCDH-NIPSNAP1 overexpression vector (**D**) were co-transfected with 3*Flag-SOD2. Thereafter, cell lysates were subjected to immunoprecipitation (IP) with anti-Flag antibodies followed by Western blot analysis against Flag, and generalized antibodies recognizing acetylated lysines, phosphorylated tyrosine (p-Tyr) or phosphorylated tyrosine/serine residues (p-Ser/Thr), respectively. Input samples were analyzed in parallel to confirm knockdown and overexpression efficiency, respectively. **E** HCT116 cells with knockdown (left) or overexpression of NIPSNAP1 (right) as per **C**, **D** were analyzed by Western blot to measure the levels of K68 and K122 acetylated SOD2. **F** HCT116 cells transduced with pCDH or pCDH-NIPSNAP1 were co-transfected with 3*Flag-SOD2 before culture with or without NAM (nicotinamide, 10 mM) or TSA (Trichostatin A, 1 μM). Thereafter, cell lysates were subjected to immunoprecipitation (IP) with anti-Flag antibodies followed by Western blot analysis against Flag, and generalized antibodies recognizing acetylated lysine. **G** HCT116 cells transduced with sh-SIRT2 or sh-SIRT3 or sh-SIRT4 or sh-SIRT5 or sh-SIRT7 in combination with pCDH-NIPSNAP1 were subjected to Western blot analysis against NIPSNAP1, SIRT isoforms or P27, respectively. **H**, **I** HCT116 cells transduced with sh-SIRT3 in combination with pCDH-NIPSNAP1 were subjected to Western blot analysis against NIPSNAP1, SIRT3, K68 and K122 acetylated and total SOD2, respectively (**H**) or to flow cytometric analysis of ROS levels (**I**). **J** Immunoprecipitation (IP) analyses undertaken in HCT116 cells examining interactions between endogenous NIPSNAP1 and SIRT3. **K**, **L** Immunoprecipitation (IP) analyses undertaken in HCT116 cells examining the effect of NIPSNAP1 knockdown (**K**) and overexpression (**L**) on interactions between SIRT3 and SOD2. HCT116 cells as per **C** and **D** were subjected to IP with anti-Flag antibodies followed by Western blot analysis to detect SOD2-3*Flag (anti-Flag) and SIRT3. Input samples were analyzed in parallel to confirm the effects of NIPSNAP1 knockdown and overexpression, respectively, and equal input levels of SIRT3. **B** Is mean ± SD; n = 3 independent experiments, two-tailed Student’s t test, Images in **A** and **C**–**L** represents three independent experiments (*P < 0.05; **P < 0.01, ***p < 0.001)

We speculated that NIPSNAP1 influenced the activity of SOD2. Indeed, repeating the experiment in two independent lines (HCT116 and HepG2 cells) confirmed that NIPSNAP1 knockdown did not affect SOD2 protein expression (Fig. 7A) but did significantly diminish SOD2 activity (Fig. 7B), proposing that NIPSNAP1 binding serves to activate SOD2. The activity of superoxide dismutases is generally known to be regulated by post-translational modifications including phosphorylation and acetylation [61–63]. On this basis, we broadly examined the phosphorylation and acetylation of SOD2 status using generalized antibodies recognizing acetylated lysines together with antibodies recognizing phosphorylated serine/threonine and tyrosine residues. Notably, we observed increased acetylation signals in Flag-tagged SOD2 after knockdown of NIPSNAP1, and conversely, decreased acetylation after NIPSNAP1 overexpression but with no discernable changes in phosphorylation (Fig. 7C, D, respectively). Moreover, it has been reported that the acetylation of SOD2 at K68 and K122 functions to inhibit its activity [63, 64] and we further interrogated acetylation changes using site specific antibodies. We found that endogenous SOD2 acetylation levels at both K68 and K122 were increased after NIPSNAP1 knockdown whereas the overexpression of NIPSNAP1 decreased their respective levels (Fig. 7E), proposing that NIPSNAP1 enhances SOD2 activity by inhibiting its acetylation.

Deacetylases can be divided into the zinc ion-dependent HDAC family and the NAD-dependent sirtuin (SIRT) family [65]. We expanded our experiments to compare treatments with the HDAC family inhibitor trichostatin A (TSA) and the SIRT family inhibitor nicotineamine (NAM). Instructively, SOD2 acetylation was increased after the addition of NAM but not TSA, and moreover, NAM reversed the decreases in SOD2 acetylation caused by the overexpression of NIPSNAP1 (Fig. 7F), suggesting that SIRTs participate in the deacetylation of SOD2 mediated by NIPSNAP1. Further dissection of the SIRT family (SIRT2, -3, -4, -5 and -7) revealed that only SIRT3 knockdown was able to reverse the decline in increased P27 levels resulting from the overexpression of NIPSNAP1 (Fig. 7G). Consistent with the notion that SIRT3 cooperates with NIPSNAP1 to inhibit SOD2 acetylation, SIRT3 knockdown increased K68 and K122 SOD2 acetylation levels while also preventing acetylation decreases in SOD2 following the overexpression of NIPSNAP1 (Fig. 7H). Consistently, the intracellular levels of ROS tracked with the acetylation status of SOD2 with high ROS levels associated with SIRT3 knockdown partially reversed following ectopic NIPSNAP1 expression (Fig. 7I).

Having established the functional interplay between NIPSNAP1 and SIRT3 in mediating SOD2 acetylation, we lastly considered how this is achieved. As the overexpression of NIPSNAP1 did not affect the expression of SIRT3 (Fig. 7G) we investigated the alternative possibility that SOD2 regulation is fundamentally based on binding events. Intriguingly, it has been reported that SOD2 activity is enhanced by SIRT3 binding [66] and notably, using immunoprecipitation assays we found that SIRT3, like SOD2, can also bind to NIPSNAP1 (Fig. 7J). However, it was unclear if NIPSNAP1 functions primarily to sequester SIRT3 or whether it acts cooperatively as part of a ternary complex. To resolve this point, we examined how NIPSNAP1 expression affects interactions between SOD2 and SIRT3. Noticeably, the overexpression of NIPSNAP1 increased binding between SOD2 and SIRT3 while in contrast, NIPSNAP1 knockdown reduced their binding (Fig. 7K, L, respectively). These data provided definitive evidence that NIPSNAP1 inhibits SOD2 acetylation by regulating the binding between SOD2 and SIRT3.

### NIPSNAP1 promotes cancer growth

**Fig. 8:**
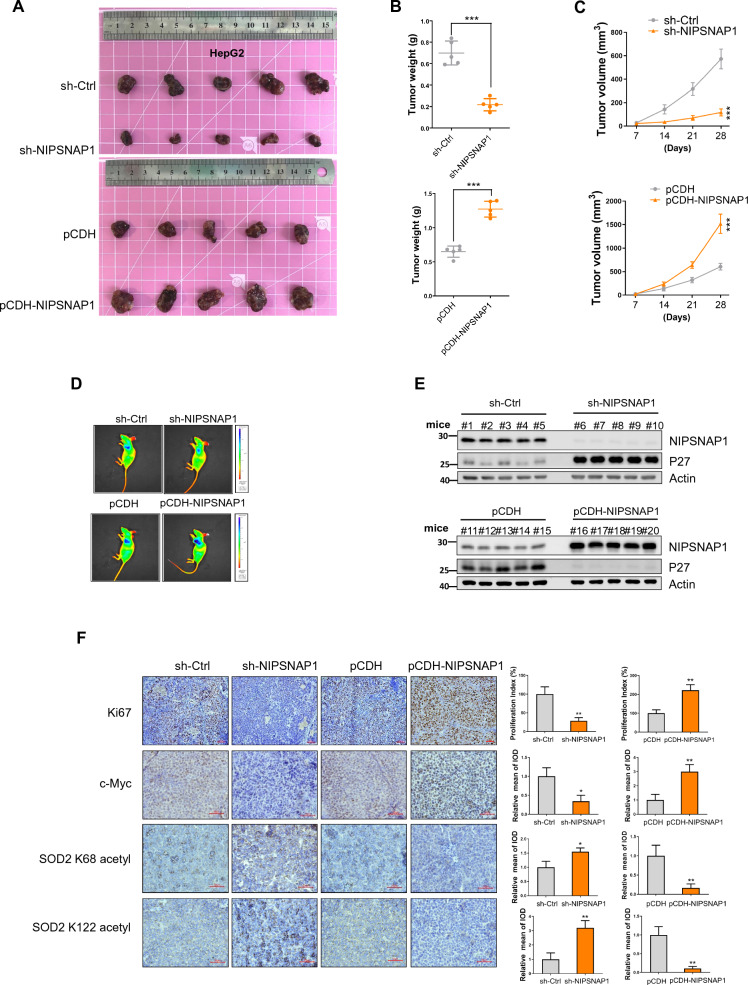
NIPSNAP1 promotes cancer growth in vivo. **A**–**D** Visual (**A**) and weight comparisons (**B**) of xenografts established over 4 weeks using HepG2 cells expressing sh-Ctrl versus sh-NIPSNAP1 (top) or pCDH versus pCDH-NIPSNAP1 (bottom). Tumor volumes were measured at the indicated time points (**C**) and with images showing representative in vivo luciferase imaging at 4 weeks (**D**) prior to humane sacrifice of the mice. **E** Western blotting analysis of lysates from the excised xenografts against NIPSNAP1, P27 and an actin loading control. **F** Photomicrographs of xenograft sections from **A** showing immunohistochemical (IHC) staining against Ki67, c-Myc, and K68 and K122 acetylated SOD2 (left). Stained sections were subjected to image analysis to calculate the relative proliferative index from Ki67 staining or alternatively the relative mean of integrated option density (IOD) (right). **B**, **C** and **F** is mean ± SD; n = 3 independent experiments, two-tailed Student’s t test, images in **A**, **D** and **E** represents three independent experiments (*P < 0.05; **P < 0.01, ***p < 0.001)

**Fig. 9:**
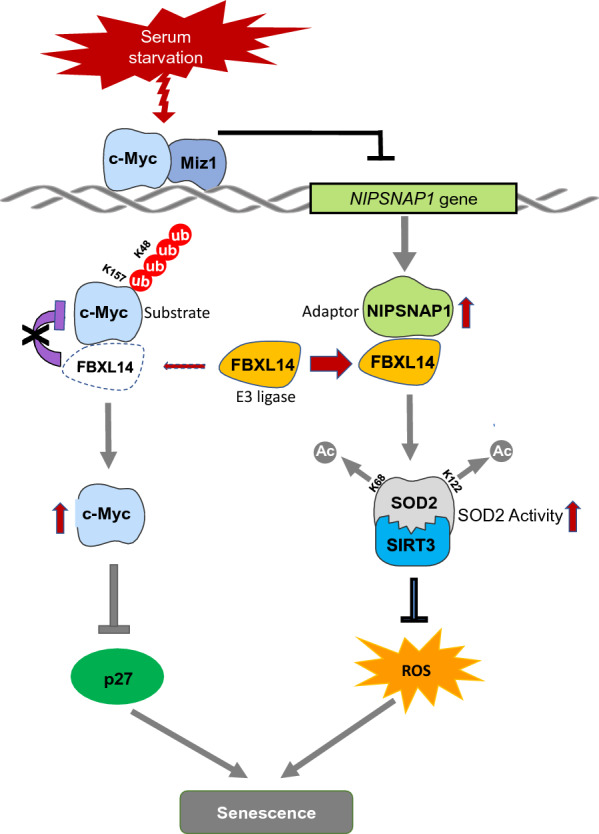
Model for NIPSNAP1-mediated regulation of cellular senescence. Schematic illustration of the proposed model depicting the dual mechanisms whereby NIPSNAP1 suppresses cellular senescence

Finally, we examined the projected clinical impact of NIPSNAP1 on cancer progression by investigating the effects of NIPSNAP1 expression on the growth of tumor xenografts. Towards this, HepG2 cells bearing luciferase in conjunction with either stable knockdown or overexpression of NIPSNAP1 along with appropriate control cells were used to establish subcutaneous tumors in the axilla of nude mice. After four weeks, comparison of final tumor weights together with tracking of tumor volumes indicated that NIPSNAP1 knockdown significantly reduced xenograft growth relative to the controls whereas ectopic NIPSNAP1 expression resulted in significantly increased growth (Fig. 8A–C). Consistently, whole body imaging at the conclusion of the experiment revealed similar differences in tumor sizes (Fig. 8D). Parallel measurements of the tumors by Western blot revealed that P27 was significantly upregulated in xenografts following NIPSNAP1 knockdown while conversely, its expression was downregulated in tumors with NIPSNAP1 overexpression (Fig. 8E). Additional analysis by immunohistochemistry confirmed that the expression of c-Myc was downregulated in tumors formed by NIPSNAP1 knockdown cells while its relative expression being increased in tumors with NIPSNAP1 overexpression (Fig. 8F). Moreover, the proliferative indices in each tumor group determined from Ki67 staining tracked with the relative expression of c-Myc. Finally, the assessment of SOD2 acetylation showed reciprocal changes with decreases and increases in K68 and K122 acetylation, respectively, accompanying NIPSNAP1 knockdown while the converse was true for tumors with NIPSNAP1 overexpression. Our working model illustrating this mechanism is shown in Fig. 9.

## Discussion

With its widespread alteration in various human cancers, c-Myc is inarguably placed as one of the most important oncogenes. Indeed, the resulting abnormal activation of cellular signaling pathways associated with increased cell proliferation plays a crucial role in the development and progression of many cancers [67–69]. Moreover, while excessive oncogenic signaling often triggers senescence, for example, as occurs with mutated N-RAS and B-RAF [70, 71], c-Myc activation promotes cell proliferation while inhibiting cellular senescence through its regulation of cell cycle-related proteins. Regarding the latter, c-Myc enjoys a specialized relationship with P27, a negative regulator that can induce cell cycle arrest and induce cellular senescence [39]. It has been shown that P27 expression is increased in c-Myc deficient cells [72, 73] with c-Myc shown to modulate the expression of P27 via different modalities. For example, c-Myc not only represses the transcription of P27 [15, 74] but it also blocks PTBP1-mediated transcription of P27 through OVAAL [48], also inhibiting the stability of P27 via Skp2 [75]. Nonetheless, c-Myc also prevents spontaneous senescence by other means such as maintaining the length of chromosomal telomeres [18, 76, 77]. This report now provides another means whereby c-Myc permits cancer cells to defeat senescence programming. When challenged by serum withdrawal, a proxy treatment for growth factor deprivation [78], the induction of NIPSNAP1 serves to maintain c-Myc expression and resist cell cycle arrest via P27-dependent mechanisms. The generalized importance of this mechanism is further emphasized by our finding that depleting NIPSNAP1 under normal conditions was itself sufficient to induce cellular senescence.

Our further dissection of the relationship between NIPSNAP1 and c-Myc uncovered their respective involvement in a mutual feedback loop. Foremost, we observed that c-Myc expression was dependent on NIPSNAP1 enhancing the stability of c-Myc by blocking its Lys48-linked ubiquitylation and subsequent proteasomal degradation. This regulatory event is based on the ability of NIPSNAP1 to selectively intervene with the activity of FBXL14. Interestingly, FBXL14 catalyzed the ubiquitylation of c-Myc at lysine 157, a site to our best knowledge not previously found to be ubiquitylated, but with prior reports of this residue being modified by acetylation [79]. Moreover, we found NIPSNAP1 acts through sequestering FBXL14, binding to both its amino and carboxyl terminal domains, thereby preventing its activity against c-Myc. Conversely, we established that the transcriptional status of NIPSNAP1 was dependent upon the repressive activity of c-Myc-Miz1, with de-repression occurring following serum withdrawal resulting in the transactivation of *NIPSNAP1*. Notably, antagonizing the induction of NIPSNAP1 was revealed to have important consequences regarding cell fate wherein cancer cells not only decreased their proliferation but were triggered to enter senescence through increases in the expression of P27. Intriguingly, NIPSNAP1 was also found to contribute to the induction of P27 expression, but in a manner that involved the homeostasis of cellular ROS levels.

ROS are products of normal cellular metabolism but the inability of scavenging mechanisms to control ROS levels leads to redox imbalances and oxidative stress injuries which can induce critical cell responses including autophagy, apoptosis, necrosis along with cellular senescence [53–55]. It is well known that excessive ROS upregulates P27 expression [56, 57] and we confirmed in our model systems that ROS activation promoted increases in P27 expression leading to cellular senescence. We found that depleting NIPSNAP1 levels was able to increase cellular ROS levels and upregulate P27 expression, proposing that NIPSNAP1 prevents cellular senescence through effects on ROS. SOD2 is the main ROS-scavenging enzyme of the cell and the only antioxidant enzyme indispensable for maintaining normal cell development and function [80, 81]. In addition to acting as a protective enzyme, SOD2 regulates intracellular redox signaling by controlling the O^2**.**−^/H_2_O_2_ ratio [63]. We found that NIPSNAP1 functions as a regulator of posttranslational modifications that govern the activity of SOD2. In particular, the competitive binding of NIPSNAP1 to SIRT3 prevents its acetyl modification of SOD2, thus maintaining SOD2 in an active state and keeping ROS below critical levels. It is further pertinent to consider that among SOD isoforms, SOD2 is exclusively expressed in mitochondria [82] and that SIRT3 is primarily a mitochondrial resident protein [83]. This implies that the involvement of NIPSNAP1 in regulating SOD2 activity also occurs within mitochondria and moreover, that imbalances in mitochondrial ROS are responsible for the NIPSNAP1 knockdown phenotypes. We did not formally establish this occurrence but given that NIPSNAP1 has been reported to be present in mitochondria [34], this appears as the most likely scenario. It also remains to be determined how the reported function of NIPSNAP1 in mitophagy [34] intersects with the senescence phenotype encountered in our study. Notwithstanding this point, we observed that NIPSNAP1 and FBXL14 were generally colocalized in the cytoplasm, suggesting their interactions occur in the cytoplasmic space. Thus, the dual mechanisms involving NIPSNAP1 appears to be played out in discrete locations, namely both mitochondria and the cytoplasm.

Lastly, it must be considered what are the broader implications of our findings. Specifically, we reveal a novel mechanism regulating the expression of c-Myc in cancer cells linked to serum deprivation, i.e., a stress state frequently encountered by cancer cells within the tumor microenvironment. Despite c-Myc representing a priority molecule in the oncology field, extensive efforts to target c-Myc, for example, with small molecule inhibitors have been frustrated by a lack of effectiveness in targeting its transcriptional mechanisms [84, 85]. Consequently, the development of anti-cancer therapies that target c-Myc remains a long-term goal, and alternative means such as indirectly targeting key effectors and co-regulators of c-Myc offer alternative possibilities. Indeed, targeting NIPSNAP1 effectively impacts c-Myc levels in cancer cells, decreases cell proliferation, and induce cell cycle arrest with accompanying induction of cellular senescence. Moreover, the second distinct NIPSNAP1 pathway involved in maintaining ROS homeostasis provides a further mechanistic opportunity to drive cancer cells into a senescent state. In this regard, the structure of NIPSNAP1 has recently been solved (unpublished; https://www.rcsb.org/structure/1vqs) and this would aid the development of suitable inhibitors. Alternatively, the rise of RNAi-based therapeutics [86] offers other opportunities to target NIPSNAP1 expression in tumors.

## Materials and methods

### Cell culture

HCT116, HepG2, including the P53 knock-out variant cells together with 293T cells were cultured in Dulbecco’s modified Eagle’s medium supplemented (Gibco) with 10% FBS (Gibco) and 1% penicillin/streptomycin (Beyotime) and maintained at 37 °C in 5% CO_2_.

### Reagents, antibodies and plasmids

The reagents and antibodies information used in this study are shown in Additional file 3: Table S1 and Additional file 4: Table S2, respectively. And the plasmid information is shown in Additional file 5: Table S3.

### RNA interference

Lentiviruses for gene knockdown or overexpression experiments were produced by transient co-transfection of 293T cells for 48 h with PLKO.1, pREV, pGag and pVSVG (2:2:2:1 ratio) or pCDH, pspax2 and pmd2.g (2:2:1 ratio). Lentiviral supernatants were filtered by 0.45um filter (Millipore) and supplemented with 8 µg/ml polybrene (Solarbio) before incubation with the target cells, followed by selection with 10 µg/ml puromycin (Solarbio). The targeting sequences used are shown in Additional file 6: Table S4.

### RNA extraction and qRT-PCR

Cells were lysed using TRIzol reagent (Invitrogen) and total RNA was extracted using chloroform and isopropanol. Complementary DNA was synthesized using the PrimeScript RT Reagent Kit (Takara) and the resultant cDNA subjected to qPCR detection with TB Green Premix Ex Taq II (Tli RNaseH Plus) (TaKaRa). Samples were amplified for 30–40 cycles using the StepOnePlus™ real-time PCR system (ThermoFisher). Comparison of relative gene expression levels to housekeeping controls (β-actin) was calculated using the 2^−ΔΔCT^ method. The specified primers sequences are listed in Additional file 6: Table S4.

### SA-β-galactosidase staining

Staining was conducted according to the manufacturers protocol provided for the SA-β-Gal Staining Kit (Beyotime). Briefly, after conducting the indicated assays in a 12-well culture plates, the cells were washed twice with PBS before adding 0.5 ml of β-Gal staining fixative solution for 15 min at room temperature. Thereafter, the cells were washed 3 times with PBS for 3 min before the addition of 0.5 ml of β-Gal staining buffer and incubated overnight at 37 °C. The resulting staining was observed by light microscopy with images recorded for quantitation.

### Western blotting

Cells were lysed on ice using lysis buffer (Beyotime) containing protease inhibitors (Roche) before dilution of equal protein amounts in SDS-PAGE Sample Loading Buffer followed by resolution using SDS-PAGE and transfer to PVDF membranes. Membranes were then blocked with 5% milk for 1.5 h and incubated with the primary antibodies overnight at 4 °C. After washing, the membranes were then incubated with secondary antibodies for 2 h at room temperature before detection of immune-complexes using ECL-based detection.

### Immunoprecipitations

After washing the cells with ice-cold PBS, 100–200 μl of lysis buffer (Beyotime) containing protease inhibitors (Roche) was added per 0.5–1 million cells. After incubation for 40 min on ice, the samples were centrifuged at 14,000×*g* for 15 min at 4 °C and the supernatant collected. 20 μl of Protein A/G Agarose beads (ThermoFisher) were pre-coupled with 100 μl of antibody working solution (diluted according to recommended concentrations) before the addition of 400 μl of lysate and incubation overnight at 4 °C. After washing three times with 700 μl of protease inhibitor-containing lysis buffer, the supernatants were removed by centrifugation and SDS-PAGE Sample Loading Buffer (2×) was added for western blot analysis.

### Immunofluorescence staining

Cells cultured on coverslips were fixed for 15 min using 4% formaldehyde solution, washed with PBS, permeabilized for 10 min using 0.4% Triton X-100. After blocking with 4% bovine serum albumin for 1 h, the samples were incubated with primary antibodies overnight at 4 °C. The next days, the samples were washed, incubated with secondary antibodies at room temperature (2 h) followed by nuclear counterstaining with DAPI (Beyotime). After mounting, the cells were imaged using a Leica TCS SP8 confocal microscope (Leica).

### Colony-formation assays

Cells were seeded into six-well plates at a density of 5000 cells/well. After two weeks of incubation, the colonies were fixed with 4% formaldehyde solution followed by staining with crystal violet. After collecting images under light microscopy, colonies were quantitated using the Analyze Particles plugin in ImageJ software.

### Chromatin immunoprecipitation (ChIP) assays

Assays were conducted according to recommendations of the ChIP kit (Beyotime). Briefly, cells were fixed by adding formaldehyde solution directly to the cell culture medium to a final concentration of 1% for 10 min at 37 °C. The cross-linking reaction was terminated by adding glycine solution, followed by washing and nuclear lysis. The resulting lysates were sonicated to generate DNA fragments with an average size of less than 1000 base pairs (bp). After immunoprecipitation with the indicated antibodies, DNA was eluted and purified for qPCR analysis. The primer sequences for CHIP are included in Additional file 6: Table S4.

### Luciferase assays

The indicated cells were co-transfected using Lipofectamine-2000 reagent (Invitrogen) with combinations of pGL3-promoter plasmids and Renilla luciferase (Additional file 7: Table S5). After 36 h, dual luciferase assays were performed according to the manufacturer’s recommendations (Promega) with firefly luciferase measurements normalized to the transfection control Renilla luciferase activity.

### Xenograft models

Five-week-old female BALB/c nude mice were purchased from Beijing Charles River Laboratory Animal Co. Ltd and permitted to acclimatize for 7 days. After randomization, 5 × 10^6^ cells in 200 μl PBS were injected subcutaneously into the axillae (n = 5 mice/group). Tumor sizes were measured weekly and analyzed using the formula (V = 0.5 × L × W^2^ Volume: V; Length: L; Width: W). After 4 weeks, the mice were sacrificed humanely and the tumors were removed and weighed. Where indicated, prior to sacrifice the mice were subjected to in vivo luciferase imaging using the IVIS Spectrum system (PerkinElmer). All animal experiments were conducted with the approval of the Animal Research Ethics Committee of Zhengzhou University (ZZU-LAC20220315[06]).

### Immunohistochemistry

After excision, a proportion of the xenografts were fixed in 4% formaldehyde before paraffin embedding using standard protocols. After cutting paraffin sections, dewaxing and rehydration, antigen retrieval was performed for 30 min before blocking with 4% BSA for 20 min and incubation with primary antibodies overnight at 4 °C. After washing, sections were incubated with diluted secondary antibodies (ORIGENE) for 30 min at room temperature. DAB solution was added to reveal the immunocomplexes before nuclear counterstaining with hematoxylin (Solarbio).

### Cell cycle analysis

Cell suspensions prepared by trypsin digestion were fixed for 12 h with ice cold 70% ethanol before aspiration of the supernatants and resuspension in propidium iodide staining solution (Beyotime) containing RNase A and further incubation at 37 °C for 30 min. Cell data were recorded by flow cytometry (BD Biosciences) and analyzed using Flowjo software.

### Cell proliferation assays

Cells were seeded in 96-well plates at 1000 cells per well and cultured for the indicated times. 10 μl of CCK-8 solution (DOJINDO) was added to each well for 1–4 h before determining absorbances (OD) at 450 nm using a microplate reader. Alternatively, the EdU Cell Proliferation Kit (Epizyme) was used according to the manufacturer’s instructions. Briefly, cells cultured in 12-well plates were pulsed with Edu before fixation and subsequent detection. Cell nuclei were counterstained stained with Hoechst 33342 and the results recording using inverted microscope (Olympus).

### Statistical analysis

All statistical analyses were performed using GraphPad Prism 8.0 software (GraphPad Software, Inc.). Statistical differences were analyzed by two-tailed Student's t-test. A p-value below 0.05 was considered statistically significant, and p-values below or equal to 0.05, 0.01, and 0.001 were denoted by *, **, and ***, respectively.

## Supplementary Information

**Additional file 1: Figure S1.** (A) Heatmap representing differentially expressed proteins in HCT116 cells cultured with or without serum (FBS) for 24 h (left) determined by mass spectrometry-based proteomic analyses. The list of the top 15 upregulated candidate proteins are shown at bottom right. (B) The knockdown efficiency of selected candidate genes from (Fig. 1A) was verified by real-time RT-PCR analysis for Fig. 1B. (C) Parallel growth assays were conducted on the cells from (Fig. 1B) over 1–4 days using CCK-8 assays. (D) The knockdown efficiency of NIPSNAP1 was verified by real-time RT-PCR analysis for Fig. 1C. (E) The successful overexpression of NIPSNAP1 was determined by real-time RT-PCR analysis for Fig. 1D. (A–E) is mean ± SD; n = 3 independent experiments, two-tailed Student’s t test (*P < 0.05; **P < 0.01, ***p < 0.001).

**Additional file 2: Figure S2.** (A) Transcription factor consensus binding sites present within the NIPSNAP1 promoter. (B) Western blotting measurements of NIPSNAP1 levels in HCT116 cells treated with sh-Ctrl, sh-SP1, sh-HIF1a, sh-c-Jun or sh-FOXO1 lentiviruses at 0, 24 and 48 h following FBS withdrawal. Each transcription factor was measured in parallel to confirm knockdown along with an actin loading control. (C) HCT116 cells were transfected with the indicated Flag-tagged wildtype (WT) or substitution mutants of c-Myc in combination with empty vector sh-Ctrl or sh-NIPSNAP1. Western blot analysis against Flag was used to detect the expression levels of c-Myc in combination with NIPSNAP1 and a GAPDH loading control. (D) Ubiquitination assays were performed in HCT116 cells by individually transfecting WT FLAG-tagged c-Myc, or the indicated substitution mutants together with HA-Ub and sh-NIPSNAP1. After immunoprecipitating c-Myc with anti-Flag, ubiquitin conjugated bands were detected using blotting against HA. (B-D) represents three independent experiments.

**Additional file 3: Table S1.** List of reagents.

**Additional file 4: Table S2.** Antibody information.

**Additional file 5: Table S3.** Plasmids.

**Additional file 6: Table S4.** Oligonucleotides used in this study.

**Additional file 7: Table S5.** Plasmid construction.

**Additional file 8: Table S6.** List of NIPSNAP1- and IgG-interacting proteins from MS analysis.

**Additional file 9: Table S7.** The LC–MS/MS results of c-Myc for analyzing its post-translational modifications, related to Fig. 4.

## Data Availability

All data generated or analyzed during this study are included in this article. Further enquiries can be directed to the corresponding author.

## Abbreviations

CHX: Cycloheximide
CHIP: Chromatin Immunoprecipitation
DAPI: 4′,6-Diamidino-2-phenylindole
FBS: Fetal bovine serum
IF: Immunofluorescence technique
IP: Immunoprecipitation
MG132: Carbobenzoxy-l-leucyl-l-leucyl-l-leucinal
ROS: Reactive oxygen species
SIRT3: Sirtuin 3
SAHF: Senescence-associated heterochromatin foci
SOD2: Manganese superoxide dismutase superoxide dismutase 2
