# Supplementary material for: NIPSNAP1 directs dual mechanisms to restrain senescence in cancer cells
Source: J Transl Med. 2023 Jun 20;21:401. doi: 10.1186/s12967-023-04232-1 (PMC10280965; doi:10.1186/s12967-023-04232-1)
Supplement: Supplementary file 1 — Additional file 1: Figure S1. (A) Heatmap representing differentially expressed proteins in HCT116 cells cultured with or without serum (FBS) for 24 h (left) determined by mass spectrometry-based proteomic analyses. The list of the top 15 upregulated candidate proteins are shown at bottom right. (B) The knockdown efficiency of selected candidate genes from (Fig. 1A) was verified by real-time RT-PCR analysis for Fig. 1B. (C) Parallel growth assays were conducted on the cells from (Fig. 1B) over 1–4 days using CCK-8 assays. (D) The knockdown efficiency of NIPSNAP1 was verified by real-time RT-PCR analysis for Fig. 1C. (E) The successful overexpression of NIPSNAP1 was determined by real-time RT-PCR analysis for Fig. 1D. (A–E) is mean ± SD; n = 3 independent experiments, two-tailed Student’s t test (*P < 0.05; **P < 0.01, ***p < 0.001). [file 12967_2023_4232_MOESM1_ESM.pdf]

A

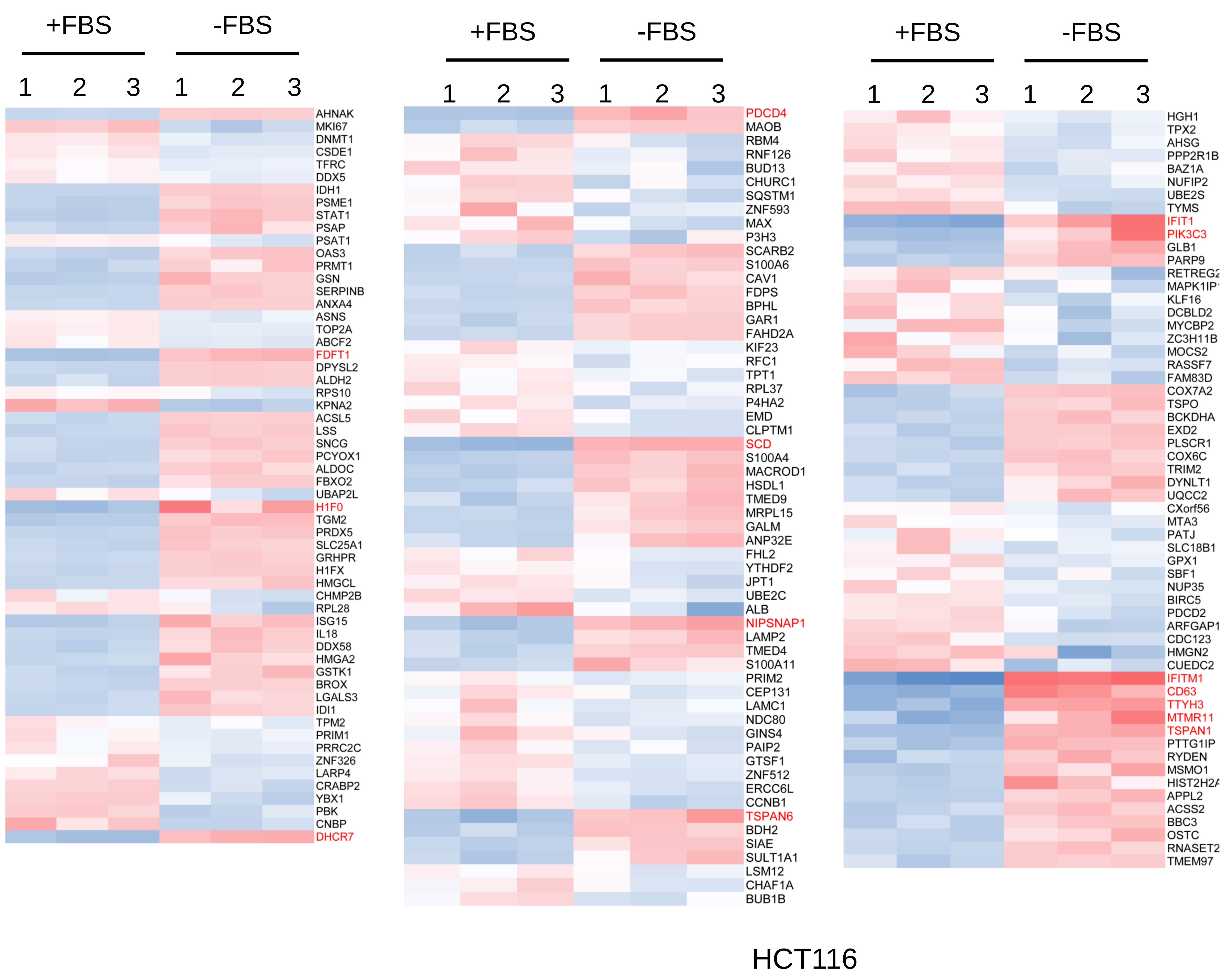

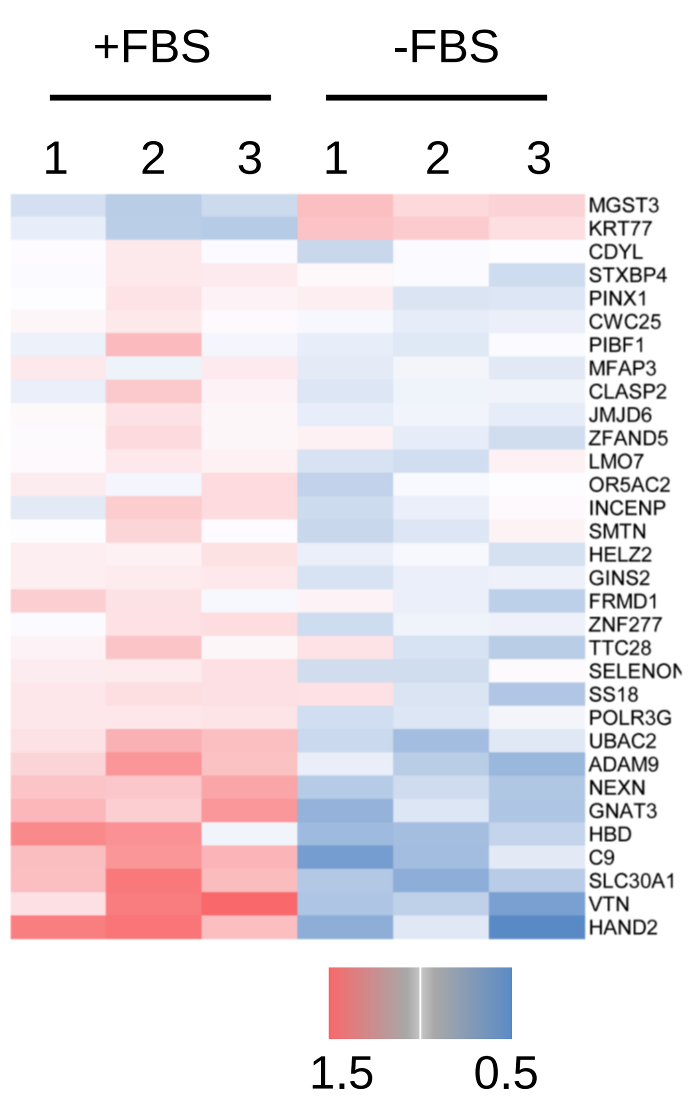

Accession	Gene Name	MW [kDa]	serum/ctrl
P13164	IFITM1	13.955326	2.389727
P09914	IFIT1	55.325223	1.772247
P08962	CD63	25.619093	1.740223
Q9C0H2	TTYH3	57.507892	1.656795
O00767	SCD	41.496143	1.570209
P07305	H1F0	20.850196	1.537352
A4FU01	MTMR11	79.495493	1.522407
O60635	TSPAN1	26.283152	1.507901
Q9UBM7	DHCR7	54.453765	1.477117
Q8NEB9	PIK3C3	101.48479	1.475
O43657	TSPAN6	27.545071	1.471456
Q9BPW8	NIPSNAP1	33.288838	1.434845
P37268	FDFT1	48.084195	1.432757
Q53EL6	PDCD4	51.703049	1.428332
P53801	PTTG1IP	20.31024	1.384554

List of top 15 upregulated candidate proteins from MS analysis of serum starvation

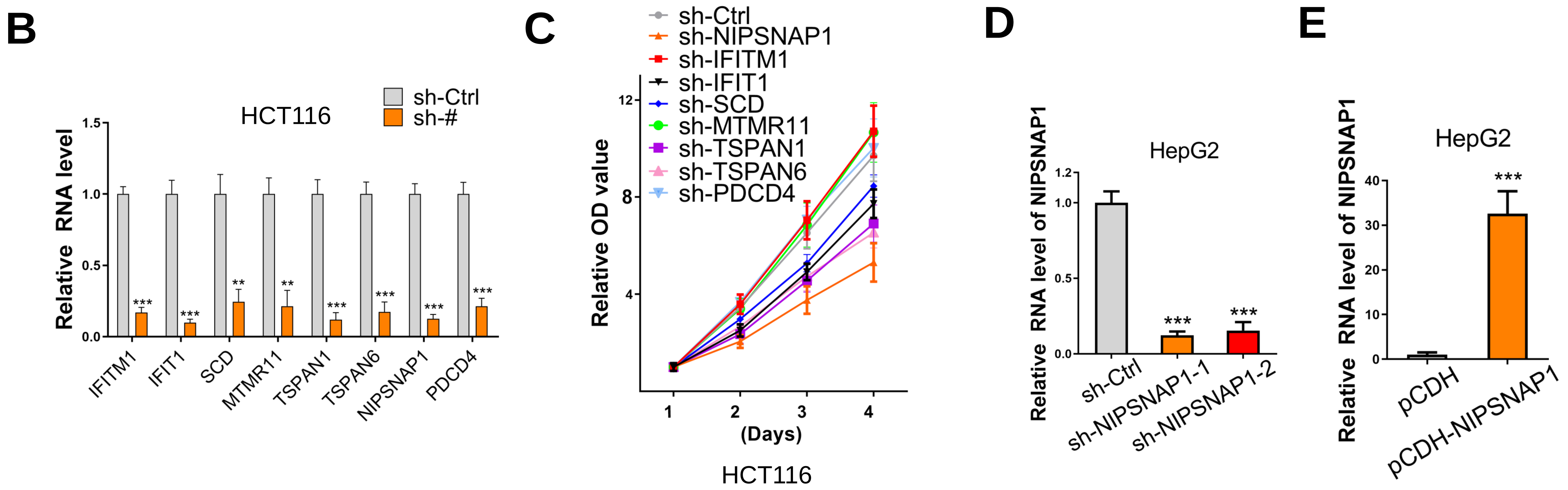

Figure S1.
