# Supplementary material for: NIPSNAP1 directs dual mechanisms to restrain senescence in cancer cells
Source: J Transl Med. 2023 Jun 20;21:401. doi: 10.1186/s12967-023-04232-1 (PMC10280965; doi:10.1186/s12967-023-04232-1)
Supplement: Supplementary file 2 — Additional file 2: Figure S2. (A) Transcription factor consensus binding sites present within the NIPSNAP1 promoter. (B) Western blotting measurements of NIPSNAP1 levels in HCT116 cells treated with sh-Ctrl, sh-SP1, sh-HIF1a, sh-c-Jun or sh-FOXO1 lentiviruses at 0, 24 and 48 h following FBS withdrawal. Each transcription factor was measured in parallel to confirm knockdown along with an actin loading control. (C) HCT116 cells were transfected with the indicated Flag-tagged wildtype (WT) or substitution mutants of c-Myc in combination with empty vector sh-Ctrl or sh-NIPSNAP1. Western blot analysis against Flag was used to detect the expression levels of c-Myc in combination with NIPSNAP1 and a GAPDH loading control. (D) Ubiquitination assays were performed in HCT116 cells by individually transfecting WT FLAG-tagged c-Myc, or the indicated substitution mutants together with HA-Ub and sh-NIPSNAP1. After immunoprecipitating c-Myc with anti-Flag, ubiquitin conjugated bands were detected using blotting against HA. (B-D) represents three independent experiments. [file 12967_2023_4232_MOESM2_ESM.pdf]

A

	Factor name	Score	Start	End	Strand	predicted site sequence
MA0079.3	SP1	13.924208	-117	-107	-1	GCTCCGCCTCC
MA0079.2	SP1	12.262342	-1729	-1720	1	CCCTGCCCCC
MA0104.4	c-Myc	11.641556	-1929	-1921	1	CACGTGGTC
MA1106.1	HIF1a	11.2201	-1429	-1420	-1	TTACGTGCTC
MA0059.1	c-Myc	10.470099	-279	-272	1	CACGAGGC
MA0480.1	FOXO1	8.845745	-1224	-1214	1	TCTTGTTGCCC
MA0099.2	c-Jun	8.680231	-502	-496	-1	TTACTCA

B

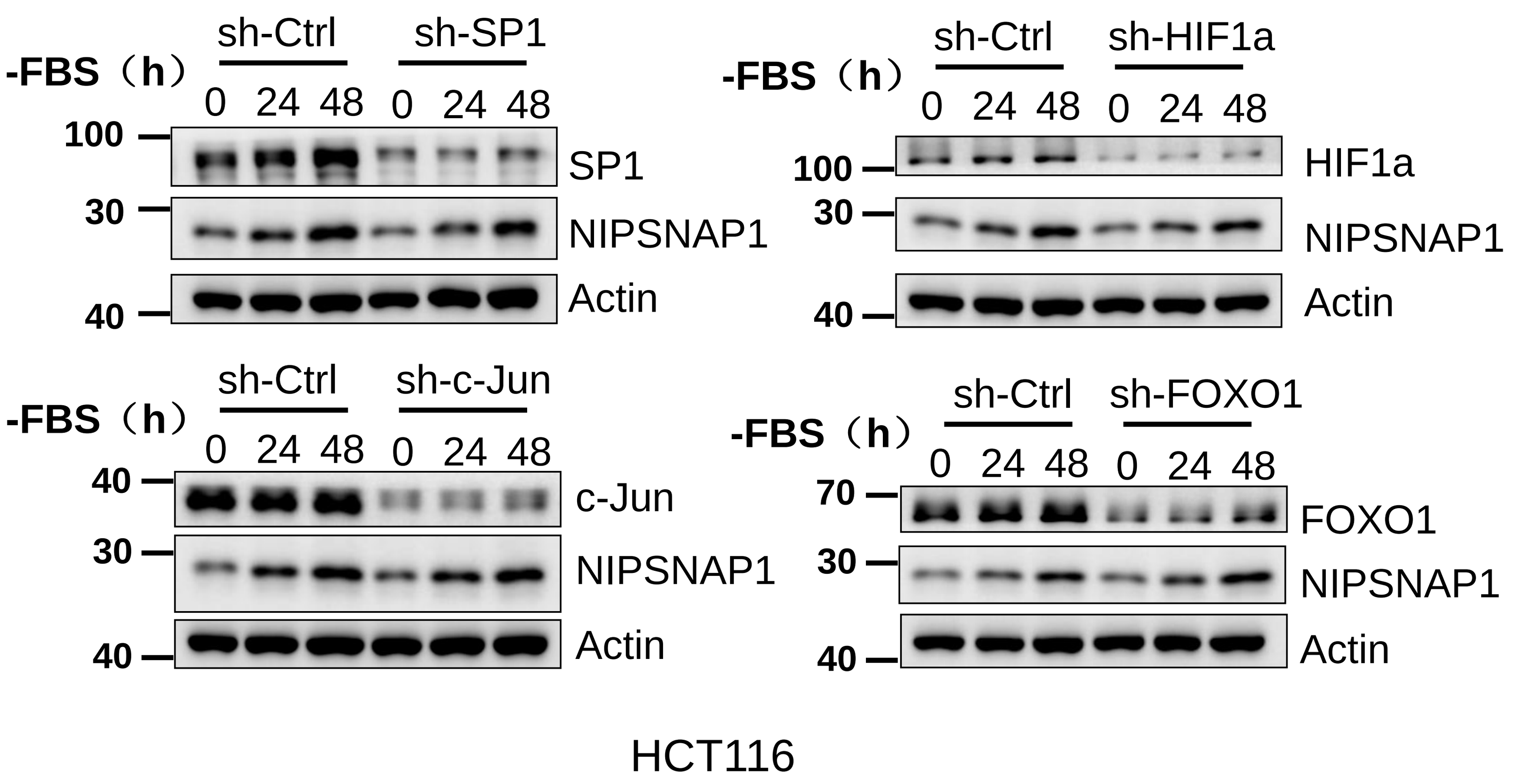

C

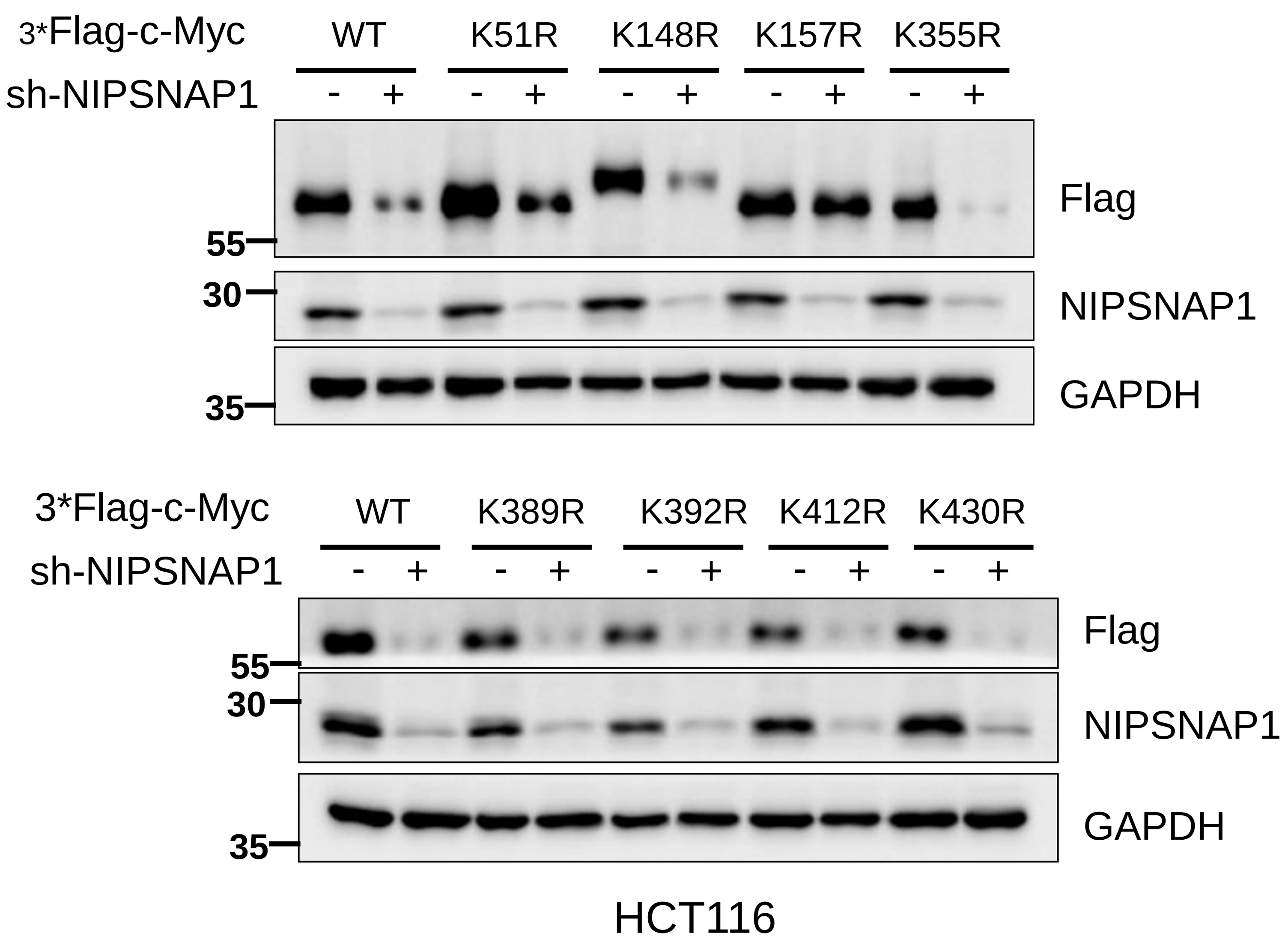

D

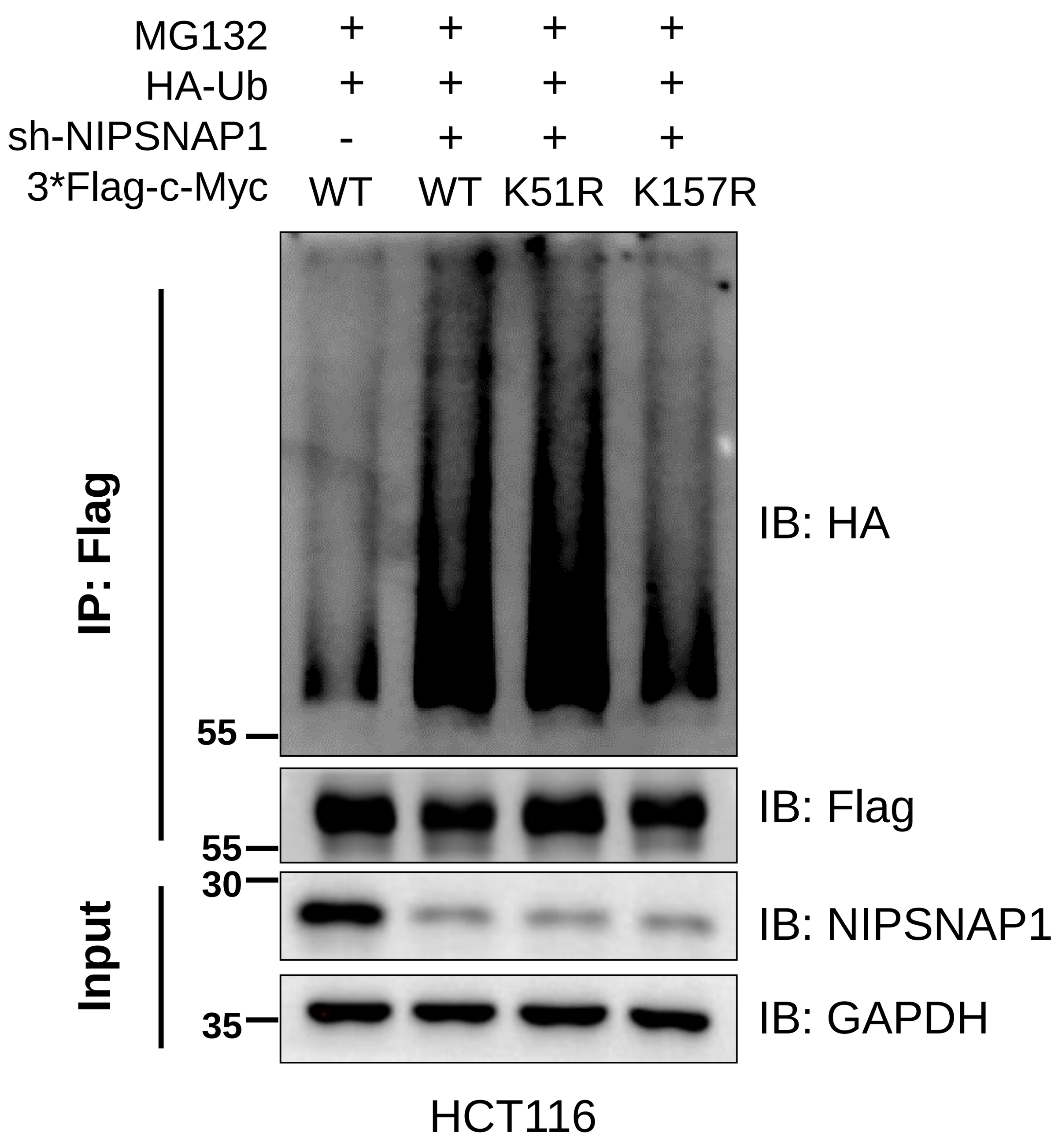

Figure S2.
