# Supplementary material for: NIPSNAP1 directs dual mechanisms to restrain senescence in cancer cells
Source: J Transl Med. 2023 Jun 20;21:401. doi: 10.1186/s12967-023-04232-1 (PMC10280965; doi:10.1186/s12967-023-04232-1)
Supplement: Supplementary file 3 — Additional file 3: Table S1. List of reagents. [file 12967_2023_4232_MOESM3_ESM.pdf]

Table S1. List of reagents

Reagent	Catalogue No.	Company
T4 DNA ligase	2011A	Takara
2xTaq PCR mix	P212-01	Vazyme
PrimeScriptTM RT reagent Kit	RR037A	Takara
SYBR Green 2xTaq mix	RR820A	Takara
Pierce Protein A/G UltraLink Resin	53133	Thermo Scientific
Dual-Luciferase Reporter Assay System	E1910	Promega
ChIP Assay Kit	P2078	Beyotime
DMEM	12800082	Gibco
TRIzol	AM9738	Invitrogen
MG132	M8699	Sigma-Aldrich
Cycloheximide	239763	Sigma-Aldrich
Lipofectamine™ 2000	11668019	Invitrogen
Anti-FLAG® M2 Magnetic Beads	M8823	Sigma-Aldrich
DAPI/Hoechst	P0131	Beyotime
EDU stainning	CX003	Epizyme
Senescence β-Galactosidase Staining kit	C0602	Beyotime
3xFlag Peptides	A6001	APExBIO
Protease Inhibitor Cocktail	4693116001	Roche
4% paraformaldehyde	P0099	Beyotime
Mut Express II Fast Mutagenesis Kit V2	C214-02	Vazyme
ROS Assay kit	S0033	Beyotime
Protease K	P1120	Solarbio Life Sciences
RIPA buffer	P0013D	Beyotime
Cell Cycle Analysis Kit	C1052	Beyotime
Cell Counting Kit-8	CK04	DOJINDO
2-Methoxyestradiol	HY-12033	MedChemExpress
Acetylcysteine	HY-B0215	MedChemExpress
Trichostatin A	HY-15144	MedChemExpress
Nicotinamide	HY-B0150	MedChemExpress
Mn-SOD activity detection kit	S0103	Beyotime
