# Supplementary material for: NIPSNAP1 directs dual mechanisms to restrain senescence in cancer cells
Source: J Transl Med. 2023 Jun 20;21:401. doi: 10.1186/s12967-023-04232-1 (PMC10280965; doi:10.1186/s12967-023-04232-1)
Supplement: Supplementary file 4 — Additional file 4: Table S2. Antibody information. [file 12967_2023_4232_MOESM4_ESM.pdf]

Table S2. Antibody Information

Antibody (Ab)	Catalogue No.	Company
c-Myc	9402	Cell Signalling Technology
NIPSNAP1	13226	Cell Signalling Technology
Ki67	ab16667	Abcam
p53	sc-126	Santa Cruz Biotechnology
p27	ab32034	Abcam
p21	P1484	Sigma-Aldrich
p15	AF5486	Affinity
p16	80772s	Cell Signalling Technology
GAPDH	60004-1-Ig	Proteintech
β-actin	AT0001	CMCTAG
SIRT2	19655-1-AP	Proteintech
SIRT4	66543-1-Ig	Proteintech
SIRT5	15122-1-AP	Proteintech
SIRT7	29729-1-AP	Proteintech
SIRT3	10099-1-AP	Proteintech
SP1	21962-1-AP	Proteintech
HIF1a	20960-1-AP	Proteintech
FOXO1	18592-1-AP	Proteintech
c-Jun	9165	Cell Signalling Technology
Miz-1	sc-136985	Santa Cruz Biotechnology
FBXL14	13934-1-AP	Proteintech
USP22	sc-390585	Santa Cruz Biotechnology
TRIM37	sc-515044	Santa Cruz Biotechnology
TRIM25	sc-166926	Santa Cruz Biotechnology
UBR5	sc-515494	Santa Cruz Biotechnology
SOD2	24127-1-AP	Proteintech
SOD2 K68 acetyl	ab137037	Abcam
SOD2 K122 acetyl	ab214675	Abcam
PRDX1	15816-1-AP	Proteintech
GPX4	67763-1-Ig	Proteintech
Ubiquitin	3936	Cell Signalling Technology
MYC tag	16286-1-AP	Proteintech
Flag tag	F1804	Sigma-Aldrich
HA tag	H9658-.2ML	Sigma-Aldrich
Anti-Mouse IgG(Alexa Fluor 488)	615-545-214	Jackson
Anti-Rabbit IgG(Alexa Fluor 633)	A-21070	Invitrogen
Anti-Rabbit IgG(Alexa Fluor 555)	A-31572	Invitrogen
Histone H3 (tri methyl K9)	ab176916	Abcam
Acetylation	66289-1-Ig	Proteintech
Phospho-Tyr	05-321	Millipore
Phospho-(Ser/Thr)	9631	Cell Signalling Technology
Anti-mouse IgG	7076	Cell Signalling Technology
Anti-rabbit IgG	7074	Cell Signalling Technology
