# Supplementary material for: NIPSNAP1 directs dual mechanisms to restrain senescence in cancer cells
Source: J Transl Med. 2023 Jun 20;21:401. doi: 10.1186/s12967-023-04232-1 (PMC10280965; doi:10.1186/s12967-023-04232-1)
Supplement: Supplementary file 5 — Additional file 5: Table S3. Plasmids. [file 12967_2023_4232_MOESM5_ESM.pdf]

Table S3. Plasmids

Plasmids	IDENTIFIER	SOURCE
pCDH	CD510B-1	SBI
pCDH-NIPSNAP1	N/A	This paper
pCDH-NIPSNAP1-Flag	N/A	This paper
pCMV3-Flag-Miz1	HG13960-NF	Sino Biological
pLKO.1-puro	SHC002	Sigma-Aldrich
pCMV-HA-Ub	P0554	MiaoLing
pCMV-HA-Ub-K48R	P8355	MiaoLing
pCMV-HA-Ub-K63R	P0855	MiaoLing
pCMV-His-UB	P4836	MiaoLing
pCMV3-FBXL14-MYC	HG25252-CM	Sino Biological
pCDNA-3×FLAG-c-MYC	P17491	MiaoLing
pCDNA-3×FLAG-c-MYC-K51R	N/A	This paper
pCDNA-3×FLAG-c-MYC-K148R	N/A	This paper
pCDNA-3×FLAG-c-MYC-K157R	N/A	This paper
pCDNA-3×FLAG-c-MYC-K355R	N/A	This paper
pCDNA-3×FLAG-c-MYC-K389R	N/A	This paper
pCDNA-3×FLAG-c-MYC-K392R	N/A	This paper
pCDNA-3×FLAG-c-MYC-K412R	N/A	This paper
pCDNA-3×FLAG-c-MYC-K430R	N/A	This paper
pECMV-3×FLAG-SOD2	P4127	MiaoLing
pGL3-NIPSNAP1-promoter	Gene synthesis	Tsingke Biotechnology
pGL3-NIPSNAP1-mutant-promoter	N/A	This paper
