# Supplementary material for: NIPSNAP1 directs dual mechanisms to restrain senescence in cancer cells
Source: J Transl Med. 2023 Jun 20;21:401. doi: 10.1186/s12967-023-04232-1 (PMC10280965; doi:10.1186/s12967-023-04232-1)
Supplement: Supplementary file 6 — Additional file 6: Table S4. Oligonucleotides used in this study. [file 12967_2023_4232_MOESM6_ESM.pdf]

Table S4. Oligonucleotides used in this study

qRT-PCR primer	F-Primer sequence (5’-3’)	R-Primer sequence (5’-3’)
β-actin	GTGGCCGAGGACTTTGATTG	CCTGTAACAACGCATCTCATATT
NIPSNAP1	GTCTCGGGAGGAGACTCGAA	GAGGCGAGATCTTCAAGGGG
FBXL14	TGCGCTCCTGTGACAACATC	TGGGCTATGTAAGCCAGACTC
c-Myc	AGCGACTCTGAGGAGGAAC	TGTGAGGAGGTTTGCTGTG
SOD2	GGAAGCCATCAAACGTGACTT	CCCGTTCCTTATTGAAACCAAGC
c-Jun	TCCAAGTGCCGAAAAAGGAAG	CGAGTTCTGAGCTTTCAAGGT
HIF1A	ATCCATGTGACCATGAGGAAATG	TCGGCTAGTTAGGGTACACTTC
IFITM1	CCAAGGTCCACCGTGATTAAC	ACCAGTTCAAGAAGAGGGTGTT
IFIT1	AGAAGCAGGCAATCACAGAAAA	CTGAAACCGACCATAGTGGAAAT
CD63	ATGCAGGCAGATTTTAAGTGCT	GTTCTTCGACATGGAAGGGATTT
TTYH3	AGAACGCTAATTTCCAGAACCC	GTGGCGAGGTATTTGGCTCTC
SCD	TTCCTACCTGCAAGTTCTACACC	CCGAGCTTTGTAAGAGCGGT
H1F0	ACTCGCAGATCAAGTTGTCCA	GGTTCGTCGCTCTTGGCTA
MTMR11	GCTGCTCAGAGTTGGTTTTGA	CCCCGAATACTGTTGGGCTT
TSPAN1	CATGCAGTTTGTCAACGTGGG	CACTTGCTCTCAGTCTTAGCAC
DHCR7	GCTGCAAAATCGCAACCCAA	GCTCGCCAGTGAAAACCAGT
PIK3C3	GTCTGGCCTAATGTAGAAGCAG	GGCAAGACGGCTCATCTGAT
TSPAN6	ACTTGTTTCAAGAGCGTTCTGC	CAATGAGCACGAAGGGGACAT
FDFT1	CCACCCCGAAGAGTTCTACAA	TGCGACTGGTCTGATTGAGATA
PDCD4	GGGAGTGACGCCCTTAGAAG	ACCTTTCTTTGGTAGTCCCCTT
PTTG1IP	GTCTGGACTACCCAGTTACAAGC	CGCCTCAAAGTTCACCCAA
**CHIP primer**	**F-Primer sequence (5’-3’)**	**R-Primer sequence (5’-3’)**
NIPSNAP1 Promoter-BS1	TAGATCCTCCTGCCTTGGCC	GCAACAGAGCAAGACCCAGT
NIPSNAP1 Promoter-BS2	CCTTGCCCCTGTCTTCACTT	ACGTTACGTGCTCCTCTGTG
LAST Promoter	GGGATCCCAGCTGACCAGCTG	GAGGCACGATGATCCAGGTGATGAG
GAPDH promoter	TACTAGCGGTTTTACGGGCG	TCGAACAGGAGGAGCAGAGAGCGA
**shRNA primer**	**Primer sequence (5’-3’)**	
sh-NIPSNAP1-1	CCGGGGCTCCCTCTTTGTTCACAAAGGATCCTTTGTGAACAAAGAGGGAGCCTTTTTG	
sh-NIPSNAP1-2	CCGGGGGGTCCCAACATCTATGAGCGGATCCGCTCATAGATGTTGGGACCCCTTTTTG	
sh-c-Myc	CCGGAACTATGACCTCGACTACGAGGATCCTCGTAGTCGAGGTCATAGTTTTTTTG	
sh-c-Jun	CCGGCGCAAACCTCAGCAACTTCAACTCGAGTTGAAGTTGCTGAGGTTTGCGTTTTTG	
sh-HIF1A	CCGGCCGCTGGAGACACAATCATATCTCGAGATATGATTGTGTCTCCAGCGGTTTTT	
sh-SP1	CCGGCCCAAGTTTATTTCTCTCTTACTCGAGTAAGAGAGAAATAAACTTGGGTTTTT	
sh-FOXO1	CCGGGCTTAGACTGTGACATGGAATCTCGAGATTCCATGTCACAGTCTAAGCTTTTTG	
sh-SOD2	CCGGGCACGCTTACTACCTTCAGTACTCGAGTACTGAAGGTAGTAAGCGTGCTTTTT	
sh-Miz1	CCGGGTGTTCACTTTAAGGCTCATACTCGAGTATGAGCCTTAAAGTGAACACTTTTT	
sh-IFITM1	CCGGGCTCTGTGACAGTCTACCATACTCGAGTATGGTAGACTGTCACAGAGCTTTTTG	
sh-IFIT1	CCGGCGTCAATGCAATTATCCATTACTCGAGTAATGGATAATTGCATTGACGTTTTTG	
sh-CD63	CCGGGCTGGCTATGTGTTTAGAGATCTCGAGATCTCTAAACACATAGCCAGCTTTTT	
sh-TTYH3	CCGGCATGAGCCAGAACGCTAATTTCTCGAGAAATTAGCGTTCTGGCTCATGTTTTTTG	
sh-SCD	CCGGCTACGGCTCTTTCTGATCATTCTCGAGAATGATCAGAAAGAGCCGTAGTTTTTG	
sh-H1F0	CCGGAGTGGCCTTCAAGAAGACCAACTCGAGTTGGTCTTCTTGAAGGCCACTTTTTTG	
sh-MTMR11	CCGGCCCAACAAGATGGCCTAGAAACTCGAGTTTCTAGGCCATCTTGTTGGGTTTTTG	
sh-TSPAN1	CCGGCCAGTCTATTAAACCCTTGATCTCGAGATCAAGGGTTTAATAGACTGGTTTTTG	
sh-DHCR7	CCGGCCCTGACTTCTGCCATAAGTTCTCGAGAACTTATGGCAGAAGTCAGGGTTTTTG	
sh-PIK3C3	CCGGCCACGAGAGATCAGTTAAATACTCGAGTATTTAACTGATCTCTCGTGGTTTTTG	
sh-TSPAN6	CCGGGCAATGTTTCTGACTCTCGTTCTCGAGAACGAGAGTCAGAAACATTGCTTTTTTG	
sh-FDFT1	CCGGCGCAACGCAGTGTGCATATTTCTCGAGAAATATGCACACTGCGTTGCGTTTTTG	
sh-PDCD4	CCGGGCGGTTTGTAGAAGAATGTTTCTCGAGAAACATTCTTCTACAAACCGCTTTTTG	
sh-PTTG1IP	TGCTGTTGACAGTGAGCGATTGCAGACTGGTGACCTTCTATAGTGAAGC<br>CACAGATGTATAGAAGGTCACCAGTCTGCAAGTGCCTACTGCCTCGGA	
sh-FBXL14	TGCTGTTGACAGTGAGCGCCGGCTGCACCCGAATCACCAATAGTGAAG<br>CCACAGATGTATTGGTGATTCGGGTGCAGCCGTTGCCTACTGCCTCGGA	
