# Supplementary material for: NIPSNAP1 directs dual mechanisms to restrain senescence in cancer cells
Source: J Transl Med. 2023 Jun 20;21:401. doi: 10.1186/s12967-023-04232-1 (PMC10280965; doi:10.1186/s12967-023-04232-1)
Supplement: Supplementary file 7 — Additional file 7: Table S5. Plasmid construction. [file 12967_2023_4232_MOESM7_ESM.pdf]

Table S5. **Plasmid construction**

Plasmids	F-Primer sequence (5'-3')	R-Primer sequence (5'-3')
pCDH-NIPSNAP1	GGAATTCATGGCTCCGCGGCTGTGCAGCAT	CGACTAGTTCACTGCAGAGGCGAGATCTTCAAG
pCDH-NIPSNAP1-Flag	GGAATTCATGGCTCCGCGGCTGTGCAGCAT	CGGCGGCCGCTTTGTCATCATCGTCCTTGTAGTCCTGCAGAGGCGAGATCTTCAAG
pCDNA-3×FLAG-c-MYC-K51R	TCTGGAGGAAATTCGAGCTGCTGCCCACCCCG	CTCGAATTTCCTCCAGATATCCTCGCTGGGCG
pCDNA-3×FLAG-c-MYC-K148R	TCGTCTCAGAGAGGCTGGCCTCCTACCAGGCTG	CAGCCTCTCTGAGACGAGCTTGGCGGCGGCCG
pCDNA-3×FLAG-c-MYC-K157R	TGCGCGCAGAGACAGCGGCAGCCCGAACCCCG	CGCTGTCTCTGCGCGCAGCCTGGTAGGAGGCC
pCDNA-3×FLAG-c-MYC-K355R	ACTGGTCCTCAGGAGGTGCCACGTCTCCACACA	ACCTCCTGAGGACCAGTGGGCTGTGAGGAGGT
pCDNA-3×FLAG-c-MYC-K389R	AATGAAAGGGCCCCCAAGGTAGTTATCCTTAA	TTGGGGGCCCTTTCATTGTTTTCCAACTCCGG
pCDNA-3×FLAG-c-MYC-K392R	CCCAGGGTAGTTATCCTTAAAAAAGCCACAGC	AGGATAACTACCCTGGGGGCCTTTTCATTGTTTT
pCDNA-3×FLAG-c-MYC-K412R	GGAGCAAAGGCTCATTTCTGAAGAGGACTTGTTGC	AAATGAGCCTTTGCTCCTCTGCTTGGACGGAC
pCDNA-3×FLAG-c-MYC-K430R	ACACAGACTTGAACAGCTACGGAACTCTTGTG	GCTGTTCAAGTCTGTGTTTCAACTGTTCTCGTCGTTT
pGL3-NIPSNAP1-mutant-promoter	GCCACCAAAAATAGTCCTAATCTTAAAATTATTATTATTTTTTTATAGA	AGGACTATTTTTGGTGGCTCATGCCTGTAATCC
